# [*b*]-Annulated Halogen-Substituted Indoles as Potential DYRK1A Inhibitors

**DOI:** 10.3390/molecules24224090

**Published:** 2019-11-13

**Authors:** Christian Lechner, Maren Flaßhoff, Hannes Falke, Lutz Preu, Nadége Loaëc, Laurent Meijer, Stefan Knapp, Apirat Chaikuad, Conrad Kunick

**Affiliations:** 1 Institut für Medizinische und Pharmazeutische Chemie, Technische Universität Braunschweig, Beethovenstraße 55, 38106 Braunschweig, Germany; 2 Zentrum für Pharmaverfahrenstechnik (PVZ), Technische Universität Braunschweig, Franz-Liszt-Straße 35A, 38106 Braunschweig, Germany; 3 Faculté de Médecine et des Sciences de la Santé UBO, 22 avenue Camille Desmoulins, 29200-Brest, France; 4 ManRos Therapeutics & Perha Pharmaceuticals, Perharidy Research Center, 29680 Roscoff, France; 5 Institute for Pharmaceutical Chemistry and Buchmann Institute for Molecular Life Sciences, Johann Wolfgang Goethe University, Max-von-Laue-Str. 9, 60438 Frankfurt am Main, Germany

**Keywords:** DYRK1A, indole, molecular docking, protein kinase inhibitor, solubility, nephelometry, X-ray structure analysis

## Abstract

Since hyperactivity of the protein kinase DYRK1A is linked to several neurodegenerative disorders, DYRK1A inhibitors have been suggested as potential therapeutics for Down syndrome and Alzheimer’s disease. Most published inhibitors to date suffer from low selectivity against related kinases or from unfavorable physicochemical properties. In order to identify DYRK1A inhibitors with improved properties, a series of new chemicals based on [*b*]-annulated halogenated indoles were designed, synthesized, and evaluated for biological activity. Analysis of crystal structures revealed a typical type-I binding mode of the new inhibitor 4-chlorocyclohepta[*b*]indol-10(5*H*)-one in DYRK1A, exploiting mainly shape complementarity for tight binding. Conversion of the DYRK1A inhibitor 8-chloro-1,2,3,9-tetrahydro-4*H*-carbazol-4-one into a corresponding Mannich base hydrochloride improved the aqueous solubility but abrogated kinase inhibitory activity.

## 1. Introduction

The dual-specificity tyrosine phosphorylation-regulated kinase 1A (DYRK1A) is the most prominent member of the DYRK group of kinases [1]. DYRK1A is involved in elementary physiological processes such as neuronal differentiation [2], apoptosis [3,4], cell cycle regulation [5], transcription regulation [6], splicing [7], EGFR stabilization [8] and synaptic processes [9]. *dyrk1a* gene is located within the Down syndrome (DS) critical region on the human chromosome 21. Individuals with trisomy 21 exhibit a 1.5-fold expression of DYRK1A compared to subjects with only two chromosome 21 copies. The characteristic symptom of Down syndrome, intellectual disability, has been linked to this overexpression of DYRK1A [10,11,12]. Overexpression of DYRK1A is also involved in the formation of the neurotoxic β-amyloid plaques and hyperphosphorylation of the tau-protein as seen in Alzheimer’s disease. In accordance with these observations, DS individuals frequently show an early onset of AD [11,12,13].

Based on the pathological consequences of increased DYRK1A activity, the enzyme has been suggested as a therapeutic drug target for treatment of DS and AD [10,11,12]. Thus, various small molecules representing a broad variety of chemotypes were described as DYRK1A inhibitors in recent years [12,14,15,16].

DYRKs belong to the CMGC kinase family and are structurally related to cyclin-dependent kinases (CDKs), mitogen-activated protein kinases (MAPKs), glycogen synthase kinases (GSKs) and cdc2-like kinases (CLKs) [17]. Since all members of the CMGC group bind ATP in their catalytic domain, the structural differences within the ATP binding site are low. The closest structural relatives of DYRK1A are DYRK1B (85% overall similarity) and CLK1 (30% overall similarity). The catalytic domain of DYRK1B differs by just one amino acid in the hinge region from DYRK1A and the binding pocket of CLK1 has a similarity of 70% compared to DYRK1A [18]. Hence, the design of selective and potent inhibitors for individual members of the CMGC group is challenging, and consequently many of the DYRK1A inhibitors published to date exhibit only a limited degree of selectivity.

**Figure 1:**
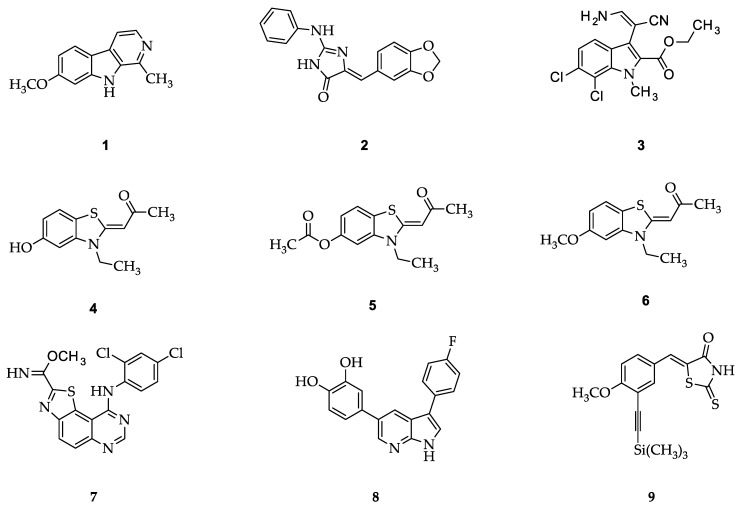
Structures of DYRK1A and/or cdc2-like kinases (CLK) inhibitors mentioned in the literature: harmine (**1**); leucettine L41 (**2**); KH-CB19 (**3**); INDY (**4**); proINDY (**5**); TG003 (**6**); EHT 5372 (**7**); F-DANDY (**8**) and FINDY (**9**).

Harmine (**1**), a β-carboline alkaloid, is a strong DYRK1A inhibitor but, due to inhibition of monoamine-oxidase A (MAO-A), is not suitable as drug candidate [19]. Leucettine L41 (**2**), derived from the marine natural product leucettamine B, is a dual DYRK1A/CLK1 inhibitor and one of the pharmacologically best profiled DYRK1A inhibitors [20,21,22,23,24,25,26]. The halogenated indole derivative KH-CB19 (**3**) also inhibits CLK1 and DYRK1A [27]. The benzothiazole derivatives INDY (inhibitor of DYRK, **4**), proINDY (**5**), and TG003 (**6**) showed a comparable inhibitory activity and selectivity profile to **1**, but **4** showed no MAO-A inhibition [28,29]. A class of DYRK1A inhibitors with remarkable potency is represented by EHT 5372 (**7**), which exhibited subnanomolar activity on DYRK1A and DYRK1B [30,31]. The DYRK1A-inhibitor F-DANDY (**8**) was reported to show efficacy in DS mice [32]. A particular mechanism of inhibition is displayed by FINDY (**9**) which is targeting the DYRK1A folding process by selective inhibition of autophosphorylation on Ser97 [33] (Figure 1).

KuFal194 (**10**) is a potent DYRK1A inhibitor (IC_50_DYRK1A = 6 nM) which displays reasonable selectivity versus DYRK1B (IC_50_DYRK1B = 600 nM) and CLK1 (IC_50_CLK1 = 500 nM). Despite a high in vitro activity of **10**, the activity in cellular DYRK1A inhibition assays (IC_50_ = 2.1 µM) was unsatisfactory [34]. The disparity between in vitro and *in cellulo* activity of **10** was explained by a low cellular uptake due to its poor physicochemical properties [35]. Representing a 7-halogenated indole derivative, the iodo-substituted indolo[3,2-c]quinoline **10** structurally resembles the dichloro-substituted inhibitor KH-CB19 (**3**).

**Figure 2:**
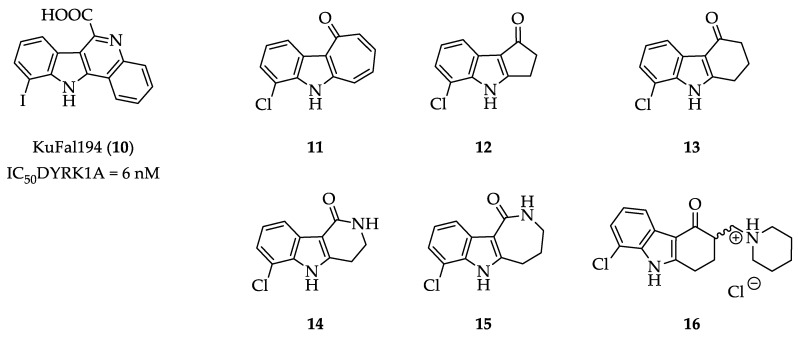
Analogues **11**–**16** of the DYRK1A inhibitor KuFal194 (**10**).

In order to improve the physicochemical properties of **10** by downsizing the structure, the [*b*]-annulated chloro-substituted indoles **11**–**15** were designed [36] (Figure 2). Although in the series of indolo[3,2-c]quinoline-6-carboxylic acids related to compound **10** the iodo-substituted analogues had shown a stronger effect, chloro-substituted compounds were prepared for the study presented here. The main reason for this was that compared to iodine, chlorine increases molar mass and lipophilicity less strongly and is also less toxicologically problematic. In addition, chlorine compounds are easier to synthesize than their iodine analogues. Prior to synthesis, the fitting probabilities of the structures to the ATP binding pocket of DYRK1A were evaluated by docking analyses, employing the published X-ray structure of **10** in DYRK1A as template [34]. For improvement of solubility, the Mannich base hydrochloride **16** was derived from **13**. Testing of these new [*b*]-annulated indoles revealed 4-chlorocyclohepta[*b*]indol-10(5*H*)-one (**11**) as a novel submicromolar dual CLK1/DYRK1A inhibitor. The binding mode of inhibitor **11** as predicted by previous docking studies was confirmed by an X-ray structure analysis of the DYRK1A-ligand complex.

## 2. Results and Discussion

### 2.1. Structure Design Considerations and Docking Analyses

**Figure 3:**
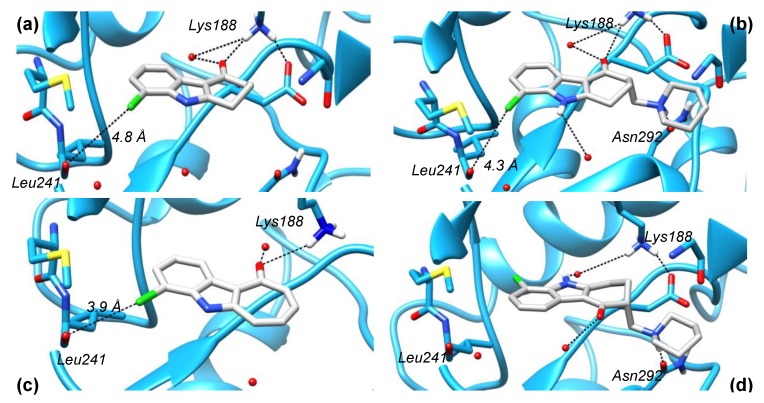
Orientations of [*b*]-annulated indoles in the ATP binding pocket of DYRK1A. (**a**) Orientation of **13** predicted by a docking analysis in DYRK1A (pdb: 4YLJ). (**b**) Orientation of (*S*)-**16** predicted by a docking analysis in DYRK1A (pdb: 4YLJ). (**c**) Result of an X-ray structure analysis of DYRK1A in complex with **11** (pdb: 6T6A). Note that the orientation of the ligand is very similar to the poses of **13** and (*S*)-**16** as predicted by the docking analyses. (**d**) Orientation of (*R*)-**16** predicted by a docking analysis in DYRK1A (pdb: 4YLJ). Note that the orientation of the tetrahydrocarbazolone ring system is flipped so that the indole nitrogen points inward and the carbonyl oxygen points outward of the binding pocket.

In all newly designed analogues, a halogen substituent was retained, since it was shown previously that such a substitution motive is important for DYRK1A inhibitory activity [34]. When the so-designed molecules **11**–**16** were docked into the structure of the DYRK1A-**10** complex (PDB:4YLJ), all congeners fitted nicely into the ATP binding pocket and displayed similar poses as the actual ligand **10**. For example, the docked cyclohexenone analogue **13** was located near the hinge region in the adenine binding area, displaying a hydrogen bond to the terminal amino group of the conserved Lys188. Similar to **10**, **13** is not forming direct hydrogen bonds to the hinge, but straightens its halogen substituent towards the backbone carbonyl oxygen of Leu241, albeit the distance of 4.8 Å between chlorine and oxygen appears too large for a halogen bond (Figure 3a). During later studies, co-crystals of the analogue **11** with the DYRK1A were produced (see below). The structure of the DYRK1A-**11** complex as established by X-ray analyses showed a similar overall orientation as predicted by the docking results for all analogues with the hydrogen bond between the carbonyl oxygen of the ligand and Lys188 as well as the chloro substituent alignment towards Leu241. In this case, the distance of halogen and oxygen is closer (3.9 Å) but still not fulfilling the requirements for strong halogen bonding (Figure 3c). Although compounds such as **11** and **13** are smaller and less lipophilic compared to the model structure **10**, they proved to be poorly soluble. To improve the solubility, a piperidinomethyl side chain was atttached to the 3-position of ligand **13**. When the resulting derivative **16** was docked into DYRK1A, only the (*S*)-isomer adopted the canonical orientation observed for the aforementioned congeners (Figure 3b). In contrast, the (*R*)-isomer displayed an orientation with a flipped tetrahydrocarbazolone motive which nevertheless fitted the ATP binding site (Figure 3d). In the docking poses, both enantiomers of **16** displayed additional hydrogen bonds between the protonated piperidine nitrogen and the backbone carbonyl oxygen of Asn292.

### 2.2. Syntheses

**Scheme 1:**
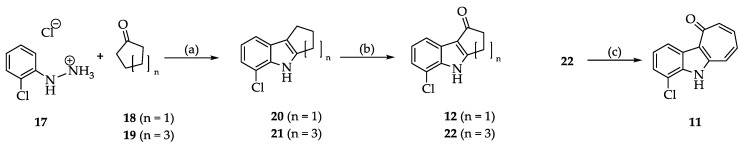
Synthesis of 4-chlorocyclohepta[*b*]indol-10(5*H*)-one (**11**) and 5-chloro-3,4-dihydrocyclopenta[*b*]indol-1(2*H*)-one (**12**). Reagents and conditions: (a) glacial acetic acid, H_2_SO_4_, 90–110 °C, 2 h, 63% (**20**) or (l)-tartaric acid, *N*,*N*’-dimethylurea (30:70), 70 °C, 3 h (21%, **21**); (b) 2,3-dichloro-5,6-dicyano-1,4-benzoquinone (DDQ), 0 °C→rt., 1–3 h, 52% (**12**) and 64% (**22**); (c) 1. PTAB, THF, rt., 48 h; 2. LiCl, DMF, reflux, 2.5 h (58%).

2-Chlorophenylhydrazine hydrochloride (**17**) and cyclopentanone (**18**) were reacted in a Fischer indole synthesis to yield the cyclopentane-annulated indole **20** [37] which upon oxidation with 2,3-dichloro-5,6-dicyano-1,4-benzoquinone (DDQ) furnished 5-chloro-3,4-dihydrocyclopenta[*b*]in-dol-1(2*H*)-one (**12**) [38]. The Fischer reaction of cycloheptanone (**19**) with 2-chloro-phenylhydrazine was carried out in a low melting mixture of (l)-tartaric acid and *N*,*N*’-dimethylurea [39] since conventional reaction conditions furnished only small yields with these starting materials. The resulting annulated indole **21** was oxidized with DDQ yielding the ketone **22**. The following aromatization of **22** with phenyltrimethylammonium tribromide (PTAB) and lithium chloride gave 4-chlorocyclohepta[*b*]indol-10(5*H*)-one (**11**) (Scheme 1). 

**Scheme 2:**
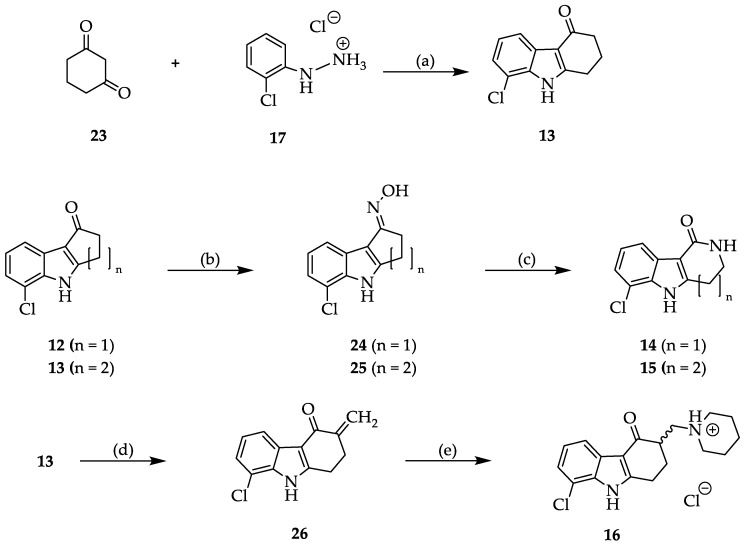
Synthesis procedures for [*b*]-annulated halogen-substituted indoles. Reagents and conditions: (a) 1. H_2_O, rt., 24 h; 2. H_2_SO_4_ (20%, *v/v*), 100 °C, 2 h (31%); (b) hydroxylamine hydrochloride (2-3 eq), sodium acetate (2-3 eq), EtOH/H_2_O, 120 °C (64% (**24**), 27% (**25**)); (c) polyphosphoric acid, 140 °C, 1 h (42% (**14**), 45% (**15**)); (d) *N*,*N*-dimethylmethylene iminium chloride, DMF, 130 °C, 2 h (74%), (e) 1. 1,4-dioxane/water (1:1), piperidine, 55 °C, 16 h; 2. propan-2-ol, HCl, Et_2_O (59%).

The cyclohexanone-annulated indole **13** was prepared by Fischer indole reaction from cyclohexane-1,3-dione (**23**) in aqueous sulfuric acid. For the preparation of lactames, the cyclic ketones **12** and **13** were converted into the oximes **24** and **25**, respectively. A subsequent Beckmann rearrangement in hot polyphosphoric acid afforded the desired ring enlarged products **14** and **15**. For the preparation of the Mannich base hydrochloride **16**, the classical reaction with formaldehyde and secondary amine afforded poor product yields. Therefore, the cyclic ketone **13** was initially converted into the α,β-unsaturated ketone **26** by reaction with *N*,*N*-dimethylmethylene iminium chloride. Nucleophilic addition of piperidine to **26** and subsequent treatment with hydrogen chloride afforded the desired salt **16** (Scheme 2).

### 2.3. Kinase Inhibitory Activity

The new compounds **11**–**16** were tested on an array of protein kinases of the CMGC family. First, the kinases were incubated with 10 µM solutions of the test compounds and the residual activities compared to controls were measured. The IC_50_ values of promising compounds were then determined from concentration-response curves (Table 1).

The results of the kinase inhibition assays show that with exception of the Mannich base **16,** all other tested derivatives are inhibitors of DYRK1A with IC_50_ values in the low micromolar or submicromolar concentration range. However, compared to the model compound **10**, the new congeners are less active by two orders of magnitude and are nonselective versus the closely related CLK1. In terms of selectivity versus other CMGC kinases, congeners **11** and **12** show a considerable selectivity versus CDKs, CK1, and GSK-3, while especially **14** and **15** appear to be rather promiscuous.

**Figure 4:**
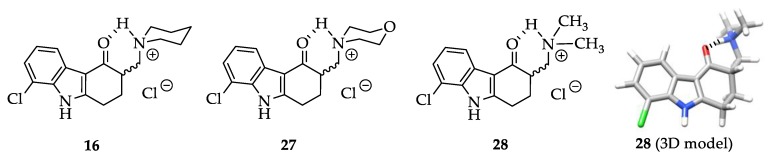
Mannich base hydrochlorides **16**, **27** and **28** displaying intramolecular hydrogen bonds; **28** also depicted as 3D model. Hashed lines represent hydrogen bonds.

A striking result was the complete loss of DYRK1A inhibitory activity observed with the Mannich base hydrochloride **16**, since the docking analyses with this molecule had predicted favorable orientations of both enantiomers in the ATP binding pocket of DYRK1A. To ensure that the piperidine structure **16** is not an inactive outlier in a series of otherwise active inhibitors, we also prepared the hydrochlorides of the morpholine and the dimethylamine analogues **27** and **28** (Figure 4)**.** These derivatives turned out to be inactive as DYRK1A inhibitors, too. The observed lack of inhibitory activity is probably explained by the fact that Mannich bases like **16**, **27,** and **28** form intramolecular hydrogen bonds involving the protonated amino group and the carbonyl oxygen. The existence of the indicated hydrogen bond becomes obvious by the diasterotopic relationship of the methyl groups in **28**, which appear in the ^1^H NMR spectrum as two distinct singlets with 3H intensity each. The puckered conformation resulting from the intramolecular hydrogen bonding is unable to fit into the ATP binding pocket of DYRK1A. Prior to accommodation of these Mannich bases to the ATP binding pocket, detachment of the intramolecular hydrogen bond would be necessary. However, the energy for breaking the intramolecular hydrogen bond is probably not sufficiently compensated by the new contacts formed upon binding of the Mannich base to the host protein.

### 2.4. Solubility

The main objective for the design and preparation of the derivatives described here was the generation of a selective DYRK1A inhibitor derived from **10** with improved solubility. Although none of the new [*b*]-annulated indoles **12**–**16** showed activity or selectivity comparable to the model compound **10**, 4-chlorocyclohepta[*b*]indol-10(5*H*)-one (**11**) was identified as a novel submicromolar dual DYRK1A/CLK1 inhibitor. In order to determine the suitability of **11** as chemical probe in biological assays, its kinetic and thermodynamic solubility [40] in aqueous phosphate buffer (pH 7.4) was evaluated and compared to both **10** and **16** (Table 2). Both KuFal194 (**10**) and **11** exhibited very poor thermodynamic solubility with saturation concentrations below the limit of quantification (< 5 µM) in the HPLC system. For standard biological assays under HTS conditions, the kinetic solubility (the solubility of the fastest precipitating polymorph) is more relevant. Regarding this parameter, **11** displayed a fivefold improved solubility compared to **10**. Although totally inactive as DYRK1A inhibitor, the Mannich base hydrochloride **16** was also investigated for solubility. As expected, the compound performed much better in both assays, exhibiting double digit millimolar solubility in the thermodynamic and single digit millimolar solubility in the kinetic assay.

### 2.5. Crystal Structure Analysis

To confirm the binding mode of the [*b*]-annulated indoles, we determined the crystal structure of DYRK1A in complex with the representative inhibitor **11** (Table 3). The binding mode of the inhibitor observed in the crystal structure was in agreement with the one suggested by our docking analyses. Specifically, the flat heterocyclic core element of **11** was located in the adenine pocket and kept in position by a hydrogen bond between its carbonyl oxygen and the ε-amino group of catalytic Lys188. The chloro substituent was directed towards the backbone carbonyl oxygen of the hinge Leu241. The latter topology suggests a weak halogen bond, although both the Cl···O distance (3.9 Å) as well as the σ-hole angle (141°) were outside or near the limits defining this non-classical interaction (< 3.27 Å and 140°–180°, respectively [41]) (Figure 3c). Limited direct contacts between the kinase and **11** suggested that the inhibitor likely achieved its potency through excellent shape complementarity.

## 3. Materials and Methods

### 3.1. General Information

The starting materials and reagents were purchased from Acros Organics (Geel, Belgium), Alfa Aesar (Karlsruhe, Germany), and Sigma-Aldrich (Steinheim, Germany). All reagents and solvents were used without further purification unless otherwise stated. Silica gel (40–63 µm) was used for purification by column chromatography. Reaction monitoring was performed using thin layer chromatography (TLC): Polygram SIL G/UV254, 0.2 mm silica gel 60, 40 × 80 mm (Macherey-Nagel, Düren, Germany), visualization by UV light (254 nm). The melting points (m.p.) were detected in open-glass capillaries on an electric variable heater (Electrothermal IA 9200, Bibby Scientific, Stone, UK). The infrared spectra were recorded on a Thermo Nicolet FT-IR 200 spectrometer (Thermo Nicolet, Madison, WI, USA) using KBr pellets. ^1^H NMR spectra and ^13^C NMR spectra were recorded on Bruker Avance III 400, Bruker Avance II 600 or Bruker Avance III HD 500 spectrometers (Bruker Biospin, Rheinstetten, Germany) (at the NMR laboratories of the Chemical Institutes of the Technische Universität Braunschweig) in DMSO-*d*_6_. Chemical shifts are reported as parts per million (ppm) relative to tetramethylsilane as internal standard (δ = 0 ppm). Signals in ^13^C spectra were assigned based on results of ^13^C DEPT135 experiments. Electron ionization (EI) mass spectra were recorded on a Finnigan-MAT 95 (Thermo Finnigan, Bremen, Germany), (EI) MS: ionization energy 70 eV. Accurate measurements were performed according to the peakmatch method using perfluorokerosene (PFK) as an internal mass reference (Department of mass spectrometry of the Chemical Institutes, TU Braunschweig, Braunschweig, German). Atmospheric pressure chemical ionization (APCI) and electrospray ionization (ESI) spectra were determined with an expression^L^ cmS spectrometer (Advion, Ltd., Harlow, UK), the APCI source was coupled with ASAP (atmospheric solids analysis probe). The ESI measurements were performed by acetic acid addition in THF or ethyl acetate via direct injection. The elemental analyses were performed on a CE Instruments Flash EA^®^ 1112 Elemental Analyzer (Thermo Quest, San Jose, CA, USA). If the elemental analysis was inconclusive a HRMS (described above) study was performed. Purity was determined using high performance liquid chromatography (HPLC) methods with isocratic or gradient elution. All compounds tested in biological systems had purity ≥95%, if not stated otherwise. The following HPLC devices and settings were used: system 1: Merck Hitachi Elite LaChrom system (Hitachi High Technologies Corporation, Tokyo, Japan) (diode array detector (DAD): L-2450; pump: L-2130; autosampler: L-2200; organizer box: L-2000); system 2: VWR Hitachi Chromaster system (Hitachi High Technologies Corporation, Tokyo, Japan) (DAD detector: 5430; column oven: 5310; pump: 5110; autosampler: 5260); column: Merck LiChroCART 125-4, LiChrospher 100 RP-18 (5 μm) (Merck, Darmstadt, Germany); system 3: Merck Hitachi Elite LaChrom system (Hitachi High Technologies Corporation, Tokyo, Japan) detector: L-2400; pump: L-2130; autosampler: L-2200; organizer box: L-2000); flow rate: 1.000 mL/min; detection wavelength: 254 nm and 280 nm ( isocratic), 254 nm (gradient); overall run time: 20 min (gradient), 7–60 min (isocratic); AUC, % method; t_ms_ = retention time, t_m_ = dead time related to DMSO. If not stated otherwise, an acetonitrile/water mixture was used for gradient elution (0–2 min: 10% ACN; 2–12 min: 10%→90% ACN (linear) 12–20 min: 90% ACN). For isocratic elution, different acetonitrile/water or acetonitrile/buffer mixtures were used. Absorption maxima (λ_max_) were extracted from the UV spectra recorded by the DAD detector in the peak maxima during HPLC runs. For the measurements of the purity, the thermodynamic solubility, and calibration with external standard of the Mannich bases, a triethylamine/triethylammonium sulfate buffer (pH 2.4) was used. For its preparation, triethylamine (20 mL) was dissolved in water (980 mL) and sodium hydroxide (242 mg) was added. The pH was adjusted to 2.4 by adding concentrated H_2_SO_4_ dropwise. The column was equilibrated with ACN/buffer (10/90) for 40 min. After that the desired ACN/buffer ratio (range 10/90–35/65) was adjusted.

### 3.2. Syntheses 

#### 3.2.1. General procedure for the synthesis of the Mannich base hydrochlorides (Procedure A)

8-Chloro-3-methylene-1,2,3,9-tetrahydro-4*H*-carbazol-4-one **26** (1 eq.) was suspended in a mixture of 1,4-dioxane/water (1:1). The amine (3 eq.) was added and stirred for the given time at the given temperature. After the reaction, the crude product was either recrystallized or extracted. For extraction ethyl acetate (30 mL) was added to the reaction mixture and extracted with aq. sulfuric acid (5%, *v/v*, 3 × 20 mL). The combined aqueous layers were alkalized with aq. 5M NaOH. The resulting basic phase was extracted with ethyl acetate (2 × 50 mL). The combined org. phases were washed with brine, water and dried over anhydrous Na_2_SO_4_. After filtration and removal of the solvent under reduced pressure, the free base (1 eq.) was dissolved in a small amount of propan-2-ol (1–5 mL). An equimolar amount of HCl in propan-2-ol (5–6 M) was added dropwise. If no precipitate formed, diethyl ether was added dropwise and the precipitate was filtered off.

#### 3.2.2. Synthesis and Characterization of **11**, **12**, **13**, **14**, **15**, **16**, **20**, **21**, **22**, **24**, **25**, **26**, **27** and **28**

*4-Chlorocyclohepta[b]indol-10(5H)-one (**11**):* To a solution of 4-chloro-6,7,8,9-tetrahydro-cyclohepta[*b*]indol-10(5*H*)-one (**22**; 83.0 mg, 0.355 mmol) in dry THF (10 mL) phenyltrimethylammonium tribromide (PTAB) (267 mg, 0.710 mmol) was added. After stirring the mixture for 28 h at room temperature, a further portion of PTAB (267 mg, 0.710 mmol) was added and stirring was continued for 20 h. A precipitate was filtered off and washed with a small portion of THF. The filtrate was evaporated and the remaining brown oil was dissolved in DMF (5 mL). Lithium chloride (48.9 mg, 1.14 mmol) was added and the mixture was refluxed under nitrogen for 2.5 h. Subsequent column chromatography (toluene-ethyl acetate 1:1) yielded a brown solid (47.4 mg, 58%). M.p.: Dec. starting at 240 °C; IR (KBr): $\tilde{v}\;\;$_max_ 3434 (NH), 1631 (C=O) cm^−1^ (Figure S3); ^1^H NMR (500 MHz, DMSO-*d*_6_) δ (ppm) = 7.07 (d, *J* = 12.2 Hz, 1H), 7.14–7.21 (m, 1H), 7.27–7.38 (m, 2H), 7.61 (d, *J* = 7.6 Hz, 1H), 7.83 (d, *J* = 10.9 Hz, 1H), 8.72 (d, *J* = 7.9 Hz, 1H), 12.77 (s, 1H, NH) (Figure S1); ^13^C NMR (126 MHz, DMSO-*d*_6_) δ (ppm) = 122.3, 122.7, 125.6, 126.4, 129.1, 133.0, 139.3 (CH), 115.6, 122.9, 127.9, 134.4, 142.0, 181.9 (C) (Figure S2); MS (APCI+): *m/z (%)* 229.8 [M + H]^+^ (100), 200.8 [M − 29]^+^ (15.5) (Figure S4); MS (APCI−): *m/z (%)* 227.8 [M − H]^−^ (100) (Figure S5); C_13_H_8_ClNO (229.66) HR-EIMS *m/z* [M]^+•^ calc. 229.02889, found 229.02923 (Figure S6); HPLC (isocr.): 99.3% at 254 nm, 99.9% at 280 nm, t_ms_ = 4.2 min, t_m_ = 1.2 min (ACN/H_2_O 40:60) (system 2) (Figure S8); HPLC (gradient): 97.9% at 254 nm, t_ms_ = 9.6 min, t_m_ = 1.2 min (system 1), λ_max_ = 220, 238, 280, 368 nm (Figure S7).

*5-Chloro-3,4-dihydrocyclopenta[b]indol-1(2H)-one (**12**):* 5-Chloro-1,2,3,4-tetrahydrocyclopenta[*b*]indole (**20**, 170 mg, 0.89 mmol) was dissolved in 1,4-dioxane/water (10:1, 11 mL) and stirred at 0 °C (ice bath). Then 2,3-dichloro-5,6-dicyano-1,4-benzoquinone (DDQ, 408 mg, 1.78 mmol) was added in small amounts. The mixture was allowed to heat up to rt. After 3 h, the precipitating solid was collected and dissolved in chloroform (30 mL) and washed with saturated aq. NaHCO_3_ (120 mL). The chloroform layer was dried over Na_2_SO_4_ and the solvent was removed under reduced pressure. After purification by column chromatography (toluene-ethyl acetate-diethylamine 2:2:1) a colorless solid (96 mg, 52%) was obtained. M.p.: 280–282 °C; IR (KBr): $\tilde{v}\;\;$_max_ 3442 (NH), 1658 cm^−1^ (C=O); ^1^H NMR (DMSO-*d*_6_, 600 MHz) δ (ppm) = 2.84–2.87 (m, 2H, CH_2_), 3.09–3.12 (m, 2H, CH_2_), 7.18 (t, 1H, *J* = 7.8 Hz, Ar-H), 7.32 (dd, 1H, *J* = 7.8, 1.0 Hz, Ar-H), 7.64 (dd, 1H, *J* = 7.8, 0.9 Hz, Ar-H), 12.40 (s, 1H, NH); ^13^C NMR (DMSO-*d*_6,_ 151 MHz) δ (ppm) = 21.1, 40.7 (CH_2_), 118.3, 122.5, 122.6 (CH), 116.2, 119.8, 122.5, 138.7, 168.6, 194.9 (C=O) (C); C_11_H_8_ClNO (205.64) calc. C 64.25, H 3.92, N 6.81, found C 64.39, H 3.89, N 6.89; HR-EIMS *m/z* [M]^+^ calc. 204.02107, found 204.02086; EIMS *m/z* (%) 205 (89), 204 (30), 177 (100); HPLC (isocr.): 100% at 254 nm, 100% at 280 nm, t_ms_ = 4.8 min, t_m_ = 1.0 min (ACN/H_2_O 30:70) (system 1) λ_max_ 239, 259, 292 nm; HPLC (gradient): 98.5% at 254 nm, t_ms_ = 8.0 min, t_m_ = 1.2 min (system 3).

*8-Chloro-1,2,3,9-tetrahydro-4H-carbazol-4-one (**13**):* 1,3-Cyclohexanedione (**23**, 561 mg, 5.00 mmol) was dissolved in water (30 mL) and 2-chlorophenylhydrazine hydrochloride (**17**, 896 mg, 5.00 mmol) was added in small amounts. The mixture was stirred for 24 h at rt. The resulting orange precipitate was filtrated and washed with water and petroleum ether. After drying the precipitate at 60 °C for 2 h, aq. sulfuric acid was added (20%, 100 mL). After the mixture reacted at 100 °C for 2 h, the solvent was removed under reduced pressure. After recrystallization from ethanol 96%, a yellow solid (340 mg, 31%) was obtained. M.p.: 266–267 °C; IR (KBr): $\tilde{v}\;\;$_max_ 3139 (N=H), 1633 cm^−1^ (C=O); ^1^H NMR (DMSO-*d*_6,_ 400 MHz) δ (ppm) = 2.13 (quint, 2H, *J* = 6.2 Hz, CH_2_), 2.43–2.48 (m, 2H, CH_2_), 3.00 (t, 2H, *J* = 6.2 Hz, CH_2_), 7.15 (t, 1H, *J* =7.7 Hz, Ar-H), 7.25 (dd, 1H, *J* = 7.7,1.0 Hz, Ar-H), 7.91 (dd, 1H, *J* = 7.7, 0.8 Hz, Ar-H), 12.19 (s, 1H, NH); ^13^C NMR (DMSO-*d*_6,_ 101 MHz): δ (ppm) = 22.7, 23.2, 37.7 (CH_2_), 119.0, 122.0, 122.7 (CH), 112.5, 115.9, 126.4, 132.8, 153.5, 193.1 (C=O) (C); C_12_H_10_ClNO (219.67) calc. C 65.61, H 4.59, N 6.38, found C 65.57, H 4.68, N 6.42; EIMS *m/z* (%) 219 (60), 191 (100), 163 (58); HPLC (isocr.): 99.1% at 254 nm, 99.1% at 280 nm, t_ms_ = 6.2 min, t_m_ = 1.0 min (ACN/buffer 30:70) (system 1) λ_max_ 240, 265, 299 nm; HPLC (gradient): 98.9% at 254 nm, t_ms_ = 9.0 min, t_m_ = 1.1 min (system 3).

*6-Chloro-2,3,4,5-tetrahydro-1H-pyrido[4,3-b]indol-1-one (**14**):* 5-Chloro-2,3-dihydrocyclopenta[*b*]in-dol-1(4*H*)-one oxime (**24**, 57 mg, 0.26 mmol) was added to PPA (4 g) at 140 °C and stirred for 1 h. After cooling to 60 °C, the mixture was poured on water (100 mL) and neutralized with aq. NaOH (2M). After extraction with ethyl acetate (3 × 50 mL) and removal of the solvent under reduced pressure, the resulting solid was purified by column chromatography (toluene-ethyl acetate-diethylamine 2:2:1). A yellow solid (24 mg, 42%) was obtained. M.p.: 284–286 °C; IR (KBr): $\tilde{v}\;\;$_max_ 3413, 3389 (NH), 1634 cm^−1^ (C=O); ^1^H NMR (DMSO-*d*_6_, 600 MHz) δ (ppm) = 2.99 (t, 2H, *J* = 6.9 Hz, CH_2_), 3.47 (dt, 2H, *J* =6.9, 2.5 Hz, CH_2_), 7.10 (t, 1H, *J* = 7.8 Hz, Ar-H), 7.13 (t, 1H, *J* = 2.4 Hz, lactam-NH), 7.19 (dd, 1H, *J* = 7.7, 1.0 Hz, Ar-H), 7.82 (dd, 1H, *J* = 7.9, 0.8 Hz, Ar-H), 11.98 (s, 1H, indole-NH); ^13^C NMR (DMSO-*d*_6,_ 151 MHz) δ (ppm) = 22.3, 39.7 (CH_2_), 118.4, 120.9, 121.6 (CH), 106.2, 115.8, 127.0,132.6, 145.6, 165.3 (C=O) (C); C_11_H_9_ClN_2_O (220.65); HR-EIMS *m/z* (%) [M]^+^ calc. 220.03979, found 220.03987; EIMS *m/z* (%) 220 (67), 191 (100), 163 (55); HPLC (isocr.): 99.2% at 254 nm, 99.8% at 280 nm, t_ms_ = 3.4 min, t_m_ = 1.0 min (ACN/H_2_O 30:70) (system 1) λ_max_ 252, 278 nm; HPLC (gradient): 94.3% at 254 nm, t_ms_ = 7.4 min, t_m_ = 1.2 min (system 3). 

*7-Chloro-3,4,5,6-tetrahydroazepino[4,3-b]indol-1(2H)-one (**15**):* 8-Chloro-1,2,3,9-tetrahydro4*H*-carb-azol-4-one oxime (**25**, 100 mg, 0.426 mmol) was added to PPA (4 g) at 140 °C and stirred for 1 h. After cooling to 60 °C, the mixture was poured on water (100 mL) and neutralized with aq. NaOH (2M). After extraction with ethyl acetate (3 × 50 mL) and removal of the solvent under reduced pressure, the resulting solid was purified by column chromatography (toluene-ethyl acetate-diethylamine 2:2:1). A yellow solid (45 mg, 45%) was obtained. M.p.: 261–262 °C; IR (KBr): $\tilde{v}\;\;$_max_ 3413, 3262 (NH), 1626 cm^−1^ (C=O); ^1^H NMR (DMSO-*d*_6_, 600 MHz) δ (ppm) = 1.97–2.03 (m, 2H, CH_2_), 3.14 (t, 2H, *J* = 6.7 Hz, CH_2_), 3.21 (dt, 2H, *J* = 5.2, 5.0 Hz, CH_2_), 7.04 (t, 1H, *J* = 7.8 Hz, Ar-H),7.16 (dd, 1H, *J* = 7.6, 0.9 Hz, Ar-H), 7.52 (t, 1H, *J* = 5.1 Hz, lactam-NH), 8.15 (dd, 1H, *J* = 8.0, 0.8 Hz, Ar-H), 11.71 (s, 1H, indole-NH); ^13^C NMR (DMSO-*d*_6,_ 151 MHz) δ (ppm) = 26.0, 28.0, 40.7 (CH_2_), 120.5, 120.9, 121.0 (CH), 107.7, 114.7, 130.4, 132.3, 143.5, 167.1 (C=O) (C); C_12_H_11_ClN_2_O (234.68); calc. C 61.41, H 4.72, N 11.94, found C 61.11, H 4.66, N 12.08; EIMS *m/z* (%) 234 (100), 191 (30), 177 (23), 163 (16); HPLC (isocr.): 100% at 254 nm, 100% at 280 nm, t_ms_ = 4.3 min, t_m_ = 1.0 min (ACN/H_2_O 30:70) (system 1) λ_max_ 281 nm.

*(R,S)-8-Chloro-3-(piperidin-1-ylmethyl)-1,2,3,9-tetrahydro-4H-carbazol-4-one hydrochloride (**16**):* According to Procedure A from 8-chloro-3-methylene-1,2,3,9-tetrahydro-4*H*-carbazol-4-one (**26**, 150 mg, 0.647 mmol) and piperidine (195 µL, 1.95 mmol) in 1,4-dioxane/water (1:1, 4 mL) for 16 h at 55 °C. After filtration, extraction and precipitation a beige solid (130 mg, 57%) was obtained. M.p.: 215–216 °C; IR (KBr): $\tilde{v}\;\;$_max_ 3420 (NH), 2619, 2515 (NH^+^), 1618 cm^−1^ (C=O); ^1^H NMR (DMSO-*d*_6,_ 500 MHz) δ (ppm) = 1.35–1.49 (m, 1H), 1.60–1.89 (m, 5H), 1.97–2.11 (m, 1H), 2.38–2.47 (m, 1H), 2.90–3.22 (m, 6H), 3.46–3.65 (m, 3H), 7.19 (t, *J* = 7.8 Hz, 1H, Ar-H), 7.29 (dd, *J* = 7.7, 1.0 Hz, 1H, Ar-H), 7.92 (dd, *J* = 7.9, 1.0 Hz, 1H, Ar-H), 9.48 (s, 1H, NH^+^), 12.39 (s, 1H, NH); ^13^C NMR (DMSO*-d_6_*, 126 MHz) δ (ppm) = 21.2, 22.0, 22.1, 22.2, 28.3, 52.5, 52.9, 56.5 (CH_2_), 41.2, 118.8, 122.3, 123.0 (CH), 111.6, 116.1, 126.3, 133.1, 153.7, 192.5 (C); C_18_H_22_Cl_2_N_2_O (353.29) calc. C 61.20, H 6.28, N 7.93 found C 60.94, H 6.34, N 7.74; MS (ESI+) *m/z* (%) 573 [M + 221]^+^ (13), 555 [M + 203]^+^ (15), 317 [M-Cl^−^]^+^ (100), 98 [M-Cl^−^ − 219]^+^ (73); HPLC (isocr.): 99.4% at 254 nm, 99.3% at 280 nm, t_ms_ = 3.3 min, t_m_ = 1.1 min (ACN/buffer 30:70) (system 1) λ_max_ 245, 266, 302 nm.

*5-Chloro-1,2,3,4-tetrahydrocyclopenta[b]indole (**20**):* 2-Chlorophenylhydrazine hydrochloride (**17**, 538 mg, 27.9 mmol) and sodium acetate (246 mg, 3.00 mmol) were dissolved in glacial acetic acid (10 mL). Cyclopentanone (**18**, 0.27 mL, 3.00 mmol) was added and the reaction mixture was stirred for 1 h at 90 °C (oil bath temperature). After addition of conc. H_2_SO_4_ (0.25 mL), the mixture was stirred for an additional hour at 110 °C. After cooling to rt, the mixture was poured into sodium acetate solution (5%, 20 mL). The precipitate was filtered off and purified by column chromatography (toluene). A colorless solid (360 mg, 63%) was obtained. M.p.: 57–59 °C; IR (KBr): $\tilde{v}\;\;$_max_ 3385 cm^−1^ (NH); ^1^H NMR (DMSO-*d*_6_, 400 MHz) δ (ppm) = 2.42–2.50 (m, 2H, CH_2_), 2.71–2.76 (m, 2H, CH_2_), 2.81–2.86 (m, 2H, CH_2_), 6.93(t, 1H, *J* = 7.7 Hz, Ar-H), 7.03 (dd, 1H, *J* = 7.7, 1.1 Hz, Ar-H), 7.28 (dd, 1H, *J* = 7.7, 0.9 Hz, Ar-H), 11.13 (s, 1H, NH); ^13^C NMR (DMSO-*d*_6_, 100.5 MHz) δ (ppm) = 24.0, 25.3, 28.2 (CH_2_), 116.8, 119.0, 119.4 (CH), 115.4, 118.7, 125.9,137.2, 145.8 (C); C_11_H_9_ClN (291.66); HR-EIMS *m/z* (%) [M]^+^^•^ calc. 190.04180, found 190.04170; EIMS *m/z* (%) 191 (99), 190 (100); HPLC (isocr.): 89.1% at 254 nm, 95.5% at 280 nm, t_ms_ = 4.2 min, t_m_ = 1.0 min (ACN/H_2_O 70:30) (system 1) λ_max_ 235, 283 nm.

*4-Chloro-5,6,7,8,9,10-hexahydrocyclohepta[b]indole (**21**):* A mixture of (l)-tartaric acid and *N*,*N*’-dimethylurea (30:70, 7.5 g) was melted at 70 °C with stirring. 2-Chlorophenylhydrazine hydrochloride (**17**, 1.79 g, 10.0 mmol) and cycloheptanone (**19**, 1.29 mL, 10.0 mmol) were added to the melt and stirring was continued at 70 °C. After 3 h, water was added and the mixture was cooled to room temperature. A resulting brown precipitate was filtered off with suction and washed with a small portion of water. Purification by column chromatography (hexane:ethyl acetate, 5:1) and crystallization (EtOH:H_2_O (7:3)) yielded a beige colored solid (470 mg, 2.14 mmol, 21%). M.p.: 99–101 °C; IR (KBr): $\tilde{v}\;\;$_max_ = 3387 (NH), 2925 (CH aliph.), 734 (Cl) cm^−1^; ^1^H NMR: (400 MHz, DMSO-*d*_6_) δ (ppm) = 1.61–1.73 (m, 4H, 2 CH_2_), 1.79–1.90 (m, 2H, CH_2_), 2.69–2.76 (m, 2H, CH_2_), 2.84–2.91 (m, 2H, CH_2_), 6.92 (t, *J* = 7.7 Hz, 1H, Ar-H), 7.02 (dd, *J* = 7.6, 0.9 Hz, 1H, Ar-H), 7.31–7.38 (m, 1H, Ar-H), 10.96 (s, 1H, NH) ppm; ^13^C NMR (101 MHz, DMSO-*d*_6_) δ (ppm) = 24.3, 27.0, 28.0, 28.4, 31.4 (CH_2_), 116.1, 119.0 (2C) (CH), 113.3, 114.9, 116.1, 119.0 (2C), 130.5, 130.9, 139.8 (C); C_13_H_14_ClN (219.71) calc. C 71.07, N 6.38, H 6.42, found C 71.08, N 6.18, H 6.37. MS (APCI+): *m/z* (%) = 220.1 [M + H]^+^ (100), 185.1 [M − 35]^+^ (59.4), 163.9 [M − 56]^+^ (46.3); MS (APCI−): *m/z* (%) = 218.1 [M − H]^−^(100); HPLC (gradient): 96% at 254 nm, t_ms_ = 13.4 min, t_m_ = 1.3 min (system 3), λ_max_ = 221, 238, 276 nm. 

*4-Chloro-6,7,8,9-tetrahydrocyclohepta[b]indole-10(5H)-one (**22**):* To a solution of 4-chloro-5,6,7,8,9,10-hexahydrocyclohepta[*b*]indole **21** (70.0 mg, 0.319 mmol, 1 eq.) in a mixture of 1,4-dioxane/water (10:1; 5.5 mL) at 0 °C was added DDQ (145 mg, 0.638 mmol) in small portions. The resulting reaction mixture was slowly allowed to warm up to room temperature. After 3 h, the resulting precipitate was filtered off with suction. The filtrate was then evaporated and chloroform (50 mL) was added to the residue. The resulting solution was washed with saturated aqueous NaHCO_3_ solution (50 mL), dried (Na_2_SO_4_), and evaporated. After purification by column chromatography (toluene-ethyl acetate 5:1), the residue was isolated as colorless solid (48 mg, 64%). M.p.: 260–264 °C; IR (KBr): $\tilde{v}\;$_max_ 3434 (NH), 1624 cm^−1^ (C=O); ^1^H NMR (DMSO-*d*_6_, 400 MHz) δ (ppm) = 1.78–1.89 (m, 2H, CH_2_), 1.89–1.97 (m, 2H, CH_2_), 2.64–2.72 (m, 2H, CH_2_), 3.14–3.22 (m, 2H, CH_2_), 7.12 (t, *J* = 7.8 Hz, 1H, Ar-H), 7.22 (dd, *J* = 7.6, 1.1 Hz, 1H, Ar-H), 8.10 (dd, *J* = 7.9, 1.0 Hz, 1H, Ar-H), 12.00 (s, 1H, NH); ^13^C NMR (DMSO-*d*_6_, 101 MHz) δ (ppm) = 21.6, 24.1, 26.6, 42.6 (CH_2_), 119.7, 121.7, 122.4 (CH), 114.6, 115.3, 129.0, 132.0, 150.4, 196.6 (C); C_13_H_12_ClNO (233.70) calc. C 66.81, N 5.99, H 5.18, found C 67.07, N 5.53, H 5.46. MS (APCI+): *m/z* (%) = 234.1 [M + H]^+^ (100), 164.0 [M − 70]^+^ (8.8), 190.0 [M − 44]^+^ (6.3); MS (APCI−): *m/z* (%) 232.1 [M − H]^−^ (100); HPLC (gradient): 99.1% at 254 nm, t_ms_ = 10.1 min, t_m_ = 1.3 min (system 3) λ_max_ 221, 240, 271, 303 nm.

*5-Chloro-2,3-dihydrocyclopenta[b]indol-1(4H)-one oxime (**24**):* 5-Chloro-2,3-dihydrocyclopenta[*b*]indol-1(4*H*)-one (**12**, 76 mg, 0.37 mmol), hydroxylamine hydrochloride (104 mg, 1.48 mmol), and sodium acetate (122 mg, 1.48 mmol) were refluxed in a mixture of ethanol (5 mL) and water (2 mL) until the starting material was no longer detectable by tlc. Silical gel (2 g) was added and the solvent was removed by evaporation. Isolation of the title compound by column chromatography (toluene-ethyl acetate-diethylamine 2:2:1) yielded pale yellow crystals (22 mg, 27%). M.p.: 248–249 °C; IR (KBr): $\tilde{v}\;\;$_max_ 3424 (NOH), 1658 cm^−1^ (C=N); ^1^H NMR (DMSO-*d*_6_, 400.4 MHz) δ (ppm) = 2.98–3.01 (m, 2H, CH_2_), 3.11–3.15 (m, 2H, CH_2_), 7.09 (t, 1H, *J* = 7.7 Hz, Ar-H), 7.20 (dd, 1H, *J* = 7.8, 1.0 Hz, Ar-H), 7.47 (dd, 1H, *J* = 7.7, 0.9 Hz, Ar-H), 10.12 (s, 1H, NOH), 11.81 (s, 1H, NH); ^13^C NMR (DMSO-*d*_6_, 100.7 MHz) δ (ppm) = 22.8, 30.8 (CH_2_), 118.1, 121.1 (2C) (CH), 116.0, 116.4, 122.6, 138.0, 155.2, 156.3 (C); C_11_H_9_ClN_2_O (220.65); calc. C 59.88, H 4.11, N 12.70; found C 59.59, H 4.04, N 12.47; MS (EI): *m/z* (%) = 220 [M]^+^^•^ (100), 203 [M − OH]^+^ (90); HPLC (isocr.): 96.7% at 254 nm and 95.3% at 280 nm, t_ms_ = 5.0 min, t_m_ = 1.0 min (ACN/H_2_O 30:70) (system 1); λ_max_: 222 nm und 258 nm; HPLC (gradient): 93.6% at 254 nm, t_ms_ = 8.3 min, t_m_ = 1.2 min (ACN/H_2_O; 0 min:10/90; 13 min:90/10; 20 min:90/10) (system 3).

*8-Chloro-1,2,3,9-tetrahydro-4H-carbazol-4-one oxime (**25**)***:** 8-Chloro-1,2,3,9-tetrahydro4*H*-carb-azol-4-one (**13**, 104 mg, 0.470 mmol), hydroxylamine hydrochloride (50 mg, 0.71 mmol), and sodium acetate (59 mg, 0.71 mmol) were refluxed in a mixture of ethanol (5 mL) and water (2 mL) until the starting material was no longer detectable by tlc. Silical gel (2 g) was added and the solvent was removed by evaporation. Isolation of the title compound by column chromatography (hexane-ethyl acetate 3:2) yielded colorless crystals (70 mg, 64%). M.p.: 214–218 °C; IR (KBr): $\tilde{v}\;\;$_max_ 3436 (NOH), 1630 cm^−1^ (C=N); ^1^H-NMR (DMSO-*d*_6_, 400.4 MHz) δ (ppm) = 1.92 (quint, 2H, *J* = 6.2 Hz, CH_2_), 2.66–2.72 (m, 2H, CH_2_), 2.84 (t, 2H, *J* = 6.2 Hz, CH_2_), 7.04 (t, 1H, *J* = 7.8 Hz, Ar-H), 7.15 (dd, 1H, *J* = 7.7, 1.0 Hz, Ar-H), 7.84 (dd, 1H, *J* = 7.7, 1.0 Hz, Ar-H), 10.38 (s, 1H, NOH), 11.55 (s, 1H, NH); ^13^C NMR (DMSO-*d*_6_, 100.7 MHz) δ (ppm) = 21.9, 22.3, 22.5 (CH_2_), 119.9, 120.8 (2C) (CH), 107.6, 115.4, 126.0, 132.8, 142.7, 152.1 (C); C_12_H_11_ClN_2_O (234.68); calc. C 61.41, H 4.72, N 11.94, found C 61.24, H 4.71, N 11.68; MS (EI): *m/z* (%) = 234 [M]^+^^•^ (100), 218 [M-O]^+^ (42), 190 [M − 44]^+^ (98), 189 [M − 45]^+^ (64); HPLC (isocr.): 99.7% at 254 nm and 99.7% at 280 nm, t_ms_ = 4.8 min, t_m_ = 1.0 min (ACN/H_2_O 40:60) (system 1); λ_max_ 234, 263 nm; HPLC (gradient): 94.2% at 254 nm, t_ms_ = 9.7 min, t_m_ = 1.1 min (ACN/H_2_O; 0 min:10/90; 13 min:90/10; 20 min:90/10) (system 3).

*8-Chloro-3-methylene-1,2,3,9-tetrahydro-4H-carbazol-4-one (**26**):* 8-Chloro-1,2,3,9-tetrahydro-4*H*-carb-azol-4-one (**13**, 1.03 g, 4.70 mmol) was dissolved in DMF (5 mL). *N*,*N*-Dimethylmethylene iminium chloride (500 mg, 5.34 mmol) was added and the solution was heated at 130 °C for 4 h. After cooling to rt., water (25 mL) was added and the precipitate was filtered off and dried to yield a beige solid (800 mg, 65%). M.p.: 225–227 °C; IR (KBr): $\tilde{v}\;\;$_max_ 3434 (NH), 1647 cm^−1^ (C=O); ^1^H NMR (DMSO-*d*_6_, 400 MHz) δ (ppm) = 2.87–2.96 (m, 2H, CH_2_), 3.05–3.12 (m, 2H, CH_2_), 5.41–5.46 (m, 1H, C=CH_2_), 5.91 – 5.95 (m, 1H, C=CH_2_), 7.19 (m, 1H, Ar-H), 7.29 (dd, *J* = 7.8, 1.1 Hz, 1H, Ar-H), 7.98 (dd, *J* = 7.7, 1.1 Hz, 1H, Ar-H), 12.34 (s, 1H, NH); ^13^C NMR (DMSO*-d*_6_, 101 MHz) δ (ppm) = 22.9, 30.6, 118.5 (CH_2_), 119.3, 122.4, 123.0 (CH), 113.1, 116.1, 126.7, 133.4, 144.11, 153.6, 182.5 (C); C_13_H_10_ClNO (231.68); HR-EIMS *m/z* [M]^+^ calc. 231.04509, found 231.04454; EIMS *m/z* (%) 231 (100), 191 (34), 163 (37), 44 (30); MS (APCI+) *m/z* (%) 232 [M + H]^+^(100), MS (APCI–): *m/z* (%) = 230 [M − H]^−^ (100); HPLC (isocr.): 96.3% at 254 nm, 96.7% at 280 nm, t_ms_ = 12.4 min, t_m_ = 1.1 min (ACN/buffer 30:70) (system 1) λ_max_ 252, 274, 320 nm.

*(R,S)-8-Chloro-3-(morpholinomethyl)-1,2,3,9-tetrahydro-4H-carbazol-4-one hydrochloride* (**27**): According to general Procedure A from 8-chloro-3-methylene-1,2,3,9-tetrahydro-4*H*-carbazol-4-one (**26**, 100 mg, 0.432 mmol) and morpholine (115 µL, 1.33 mmol) in 1,4-dioxane/water (1:1, 4 mL) for 6 h at 100 °C. After filtration, extraction and precipitation a beige solid (88 mg, 58%) was obtained. M.p.: 222–224 °C; IR (KBr): $\tilde{v}\;\;$_max_ 3429 (NH), 2458 (NH^+^), 1613 cm^−1^ (C=O); ^1^H NMR (DMSO-*d*_6,_ 500 MHz) δ (ppm) = 1.98–2.09 (m, 1H), 2.36–2.44 (m, 1H), 3.10–3.15 (m, 2H), 3.15–3.25 (m, 4H), 3.50–3.59 (m, 2H), 3.67–3.73 (m, 1H), 3.79–3.87 (m, 2H), 3.95–4.02 (m, 2H), 7.20 (t, *J* = 7.8 Hz, 1H, Ar-H), 7.30 (dd, *J* = 7.8, 1.0 Hz, 1H, Ar-H), 7.88–7.95 (m, 1H, Ar-H), 9.88 (s, 1H, NH^+^), 12.39 (s, 1H, NH); ^13^C NMR (DMSO*-d_6_*, 126 MHz) δ (ppm) = 22.1, 28.0, 51.7 (2C), 56.8, 62.9, 63.0 (CH_2_), 40.7, 118.8, 122.4, 123.1 (CH), 111.6, 116.1, 126.2, 133.1, 153.7, 192.4 (C); C_17_H_20_Cl_2_N_2_O_2_ (355.26); HR-ESIMS *m/z* (%) [[2M-2HCl + Na+]^+^ calc. 659.21623 found 659.21657, [M-HCl + Na^+^]^+^ calc. 341.10274 found 341.10254 (5), [M – Cl^−^]^+^ calc. 319.12078 found 319.12115 (100), [M-Cl − Cl^−^]^+^ calc. 285.15975 found 285.16009; MS (ESI+) *m/z* (%) 319 [M − Cl^−^]^+^ (22), 179 [M − Cl^−^-140]^+^ (71), 100 [M − Cl^−^-219]^+^ (100); HPLC (isocr.): 96.4% at 254 nm, 95.3% at 280 nm, t_ms_ = 5.2 min, t_m_ = 1.1 min (ACN/buffer 20:80) (system 1) λ_max_ 246, 266, 303 nm.

*(R,S)-8-Chloro-3-[(dimethylamino)methyl]-1,2,3,9-tetrahydro-4H-carbazol-4-one hydrochloride* (**28**): According to Procedure A with 8-chloro-3-methylene-1,2,3,9-tetrahydro-4*H*-carbazol-4-one (**26**, 150 mg, 0.647 mmol) and dimethylamine (250 µL, 1.95 mmol) in 1,4-dioxane/water (1:1, 4 mL) for 17 h at 55 °C. After filtration, extraction and precipitation a beige solid (31 mg, 15%) was obtained. M.p.: 202-203 °C; IR (KBr): $\tilde{v}\;\;$_max_ 3398 cm^−1^ (NH), 2615 cm^−1^ (NH^+^), 1634 cm^−1^ (C=O), 1618 cm^−1^ (C=C), 1473 cm^−1^ (HN-C=C-C); ^1^H NMR (600 MHz, DMSO-*d*_6_) δ (ppm) = 1.95–2.05 (m, 1H), 2.30–2.37 (m, 1H), 2.85 (s, 3H, CH_3_), 2.86 (s, 3H, CH_3_), 3.08–3.20 (m, 4H), 3.59–3.67 (m, 1H), 7.20 (t, *J* = 7.7 Hz, 1H, Ar-H), 7.30 (dd, *J* = 7.8, 1.0 Hz, 1H, Ar-H), 7.88–7.93 (m, 1H, Ar-H), 9.55 (s, 1H, NH^+^), 12.43 (s, 1H, NH); ^13^C NMR (DMSO*-d_6_*, 151 MHz) δ (ppm) = 42.6, 43.4 (CH_3_) 22.1, 27.6, 57.5 (CH_2_), 40.8, 118.7, 122.4, 123.1 (CH), 111.6, 116.1, 126.2, 133.2, 153.9, 193.2 (C); C_15_H_18_Cl_2_N_2_O (313.22); HR-ESIMS *m/z* (%) [M − Cl^−^]^+^ calc. 277.11022, found 277.11051 (100); MS (ESI+) *m/z* (%) 371 [M–HCl + 95]^+^ (30), 277 [M − Cl^−^]^+^ (100), 145 [M-HCl − 131]^+^ (20), 58 [M-HCl − 218]^+^ (64), MS (ESI−) *m/z* (%) 275 [M-Cl − 2H^+^]^-^ (100); HPLC (isocr.): 99.3% at 254 nm, 99.4% at 280 nm, t_ms_ = 4.7 min, t_m_ = 1.1 min (ACN/buffer 20:80) (system 1) λ_max_ 245, 266, 303 nm. 

### 3.3. Molecular Docking

The program GOLD [42] (version 5.2.2) on a Windows 7 system was used for docking studies. The crystal structure 4YLJ was downloaded from the protein data bank (pdb) [43]. Chain A was used as template structure because it best fulfilled the requirements for a halogen bond between the iodine atom and a water molecule. Protein preparation was performed using the Quick preparation function of MOE (Molecular Operating Environment, version 2015.1001) [44] by selecting the options “Use Structure Preparation”, “Use Protonate 3D for Protonation” and “Allow ASN/GLN/HIS Flips in Protonate 3D”, “Delete Water Molecules farther then 4.5 Å from Ligand or Receptor/Ligand”, “Tether Receptor” (Strength 10, Buffer 0.25), “Fix Atoms Farther than 8 Å from Ligands”, “Hydrogens close to Ligands will not be Fixed” and “Refine” (RMS Gradient of 0.1 kcal/mol/Å) in the QuickPrep panel were selected for refinement, protonation and energy minimization of the protein structure. The protein and ligand were protonated at pH 7. The prepared protein was saved as a mol2 file. The ligands (both stereo isomers for **16**, **27, 28**) were also created with MOE. The ligands were protonated with “Protonate 3D” (T = 300, pH = 7.4, Salt = 0.1, Electrostatics: GB/VI, Dielectric: 2, van der Waals: 800R3, Cutoff (A): 15, Solvent: 80, Cutoff (A): 10; enable disconnected metal treatment) and the conformations with the lowest energy were evaluated with “Energy Minimize” (Forcefield: Amber10:EHT, R-Field 1:80, Cutoff (8,10); Cell: No Periodicity; Charges: The System Appears Reasonable; Constraints: Rigid Water Molecules; Gradient: 0.1 RMS kcal/mol/Å^2^). The conformations with the lowest energies were evaluated and saved as mol2 files. Docking runs were performed using the wizard of GOLD in the HERMES interface (version 1.6.2) (CCDC Software Ltd., Cambridge, UK). Missing hydrogen atoms were added, and the ligands and all water molecules distant from the binding pocket were removed from the protein structure. The binding site was defined as a zone of 10 Å around the co-crystallized inhibitor. The implemented scoring function chemscore_kinase was used for evaluation and ranking of the docking results. Search efficiency was set to 200%, 10 different poses were generated, the function “generate diverse solutions” was activated and the option “allow early termination” was turned off. For the retained water molecules, the option “toggle” was used. Results of docking experiments were analyzed and visualized using UCSF Chimera, version 1.12 (Resource for Biocomputing, Visualization, and Informatics at the University of California, San Francisco, USA (supported by NIGMS P41-GM103311)) [45]. 

### 3.4. Protein Kinase Assays

Protein kinase assays with the derivatives **11**–**15** were carried out by radiometric methods using protein kinases, reagents, and conditions as described before [34]. The DYRK1A inhibitory activities of the Mannich bases **16**, **27** and **28** were assayed in 384-well plates using the ADP-GloTM assay kit (Promega, Madison, WI) according to the recommendations of the manufacturer. This assay is a luminescent ADP detection assay that provides a homogeneous and high-throughput screening method to measure kinase activity by quantifying the amount of ADP produced during a kinase reaction. Briefly, the reactions were carried out in a final volume of 6 µL for 30 min at 30 °C in appropriate kinase buffer, with either protein or peptide as substrate in the presence of 10 µM ATP. After that, 6 µL of ADP-GloTM Kinase Reagent was added to stop the kinase reaction. After an incubation time of 50 min at room temperature (rt), 12 µL of Kinase Detection Reagent was added for one hour at rt. The transmitted signal was measured using the Envision (PerkinElmer, Waltham, MA) microplate luminometer and expressed in Relative Light Units (RLU). In order to determine the half maximal inhibitory concentration (IC_50_), the assays were performed in duplicate in the absence or presence of increasing doses of the tested compounds. Kinase activities are expressed in % of maximal activity, i.e. measured in the absence of inhibitor. The peptide substrate was obtained from Proteogenix (Schiltigheim, France). RnDYRK1A-kd was assayed in buffer A with 0.033 μg/µl of the peptide KKISGRLSPIMTEQ as substrate (buffer A: 10 mM MgCl_2_, 1 mM EGTA, 1 mM DTT, 25 mM Tris-HCl pH 7.5, 50 µg/mL heparin). All data points for construction of dose response curves were recorded in triplicate. Typically, the standard deviation of single data points was below 10%.

### 3.5. Determination of Thermodynamic Solubility

For determination of thermodynamic solubility [35] an aqueous phosphate buffer at pH 7.4 with sodium chloride was used. The buffer was prepared by dissolving Na_2_HPO_4_ × 2 H_2_O (298 mg), KH_2_PO_4_ (19 mg) and NaCl (800 mg) in water (100 mL). The pH was adjusted to 7.4 by addition of aq. hydrochloric acid (3 mol/L). The thermodynamic solubility was determined using a shake-flask method with quantification by a buffer-HPLC method. The test compounds were added to a sealed Whatman miniUniPrep vial (GE Healthcare, Freiburg, Germany) until the test compound showed visual solids in aqueous phosphate buffer pH 7.4 (300 µL). The mixtures were shaken at 25 °C and 400 rpm for 24 and 48 h (IKA KS 3000 ic control, IKA-Werke, Staufen, Germany). For each time (24 and 48 h) two independent measurements at two different detection wavelengths (254 and 280 nm) were determined. After 24 h and 48 h the mixtures were inspected for remaining solids. The concentrations in the resulting saturated solutions were quantified by isocratic HPLC with external standard calibration. The equilibrium was considered to have been reached after 48 h if the concentration was comparable to the 24 h measurement. For calibration, a stock solution of the test compounds in DMSO was prepared and diluted with DMSO to suitable concentrations. The AUC at the wavelength 254 nm and 280 nm was used for quantification. If the compounds caused signals lower than the limit of quantification, the thermodynamic solubility was indicated as <5.0 µM, which was the lowest detectable concentration of the calibration solutions.

### 3.6. Determination of Kinetic Solubility

The kinetic solubility was determined using laser nephelometry [35]. A stock solution based on the results of the thermodynamic solubility measurement of the test compounds in DMSO was prepared. The stock solution was diluted with DMSO to get different concentrations. The DMSO dilutions (5 µL) were placed on a 96-well plate filled with aqueous phosphate buffer pH 7.4 (195 µL, same buffer as for thermodynamic solubility). Two dilution series of the stock solution were measured. A blank was determined for every measurement. The well plate was scanned by a nephelometer (Nephelostar Plus, BMG Labtech, Ortenberg, Germany). Precipitated particles scatter the laser light which is detected by the nephelometer. The intensity of the scattered light is assumed to be proportional to the particle concentration in the suspension. The different concentrations were plotted versus the intensities measured by the nephelometer. The resulting kick-off curves led directly to the kinetic solubility by determining the intersection of the linear straight line paralleling the x axis and the linear slope.

### 3.7. Crystallization of DYRK1A-**11** Complex, Data Collection and Structure Determination 

Crystallization of the DYRK1A-**11** complex was carried out as described previously[34], albeit with using solution containing 31% PEG 400, 0.2 M lithium sulfate and 0.1 M tris, pH 7.5. Diffraction data collected at Diamond Light Source, I04-1 were processed using Xia2 [46] and scaled with Scala [47]. Molecular replacement was performed using Phaser [48] and the coordinates of DYRK1A [34]. Model rebuilding was performed in COOT [49], alternated with refinement in REFMAC [50]. The final structure was validated for geometric correctness with Molprobity [51], and was deposited under accession code 6T6A.

## 4. Conclusions

In the search for DYRK1A inhibitors with improved solubility based on the model compound **10**, [*b*]-annulated halogenated indoles were designed, synthesized and evaluated for biological activity. Among a variety of new structures, 4-chlorocyclohepta[*b*]indol-10(5*H*)-one (**11**) was identified as a new submicromolar dual DYRK1A/CLK1 inhibitor with slightly improved kinetic solubility. A cocrystallization of **11** with DYRK1A enabled an X-ray structure analysis, which corroborated the binding mode of the ligand as predicted by previous docking studies. Mannich base hydrochlorides of the structurally related 8-chloro-1,2,3,9-tetrahydro-4*H*-carbazol-4-one (**13**) proved to be inactive as DYK1A inhibitors. 

## Figures and Tables

**Table 1:** Inhibition of protein kinases by [*b*]-annulated chloroindoles. IC_50_ values (µM) ^1^.

Entry.	CDK1	CDK2	CDK5	CDK9	CK1	CLK1	DYRK1A	GSK-3
**10**	>10	>10	>10	>10	>10	0.50	0.006	>10
**11**	>10	>10	>10	>10	>10	0.17	0.20	>10
**12**	>10	>10	>10	>10	>10	0.89	0.71	>10
**13**	n.t.	n.t.	8.2	n.t.	>10	0.30	1.6	4.4
**14**	1.2	1.7	0.81	0.88	3.6	0.12	0.19	2.7
**15**	7.0	2.5	2.5	2.2	4.2	0.32	2.2	>10
**16**	n.t.	n.t.	n.t.	n.t.	n.t.	n.t.	>10	n.t.

^1^ Results from dose response curves. Data points were recorded as triplicates. n.t. = not tested. Standard deviation of data points of single concentrations was typically below 10%. Assays were performed by radiometric methods, with the exception of **16**, which was tested using the ADP-GloTM assay kit (Promega, Madison, WI). Data for **10** taken from Falke et al. [34].

**Table 2:** Solubility data for selected [*b*]-annulated indoles.

Compound	S_exp., therm., pH 7.4_ [µM] ^a^	S_exp., kin., pH 7.4_ [µM] ^b^
KuFal194 (**10**)	<5	5.28 (4.99–5.57)
**11**	<5	26.1 (25.6–26.5)
**16**	13,650 (11,100–16,200)	2310 *

^a^ Thermodynamic solubility (phosphate buffer pH 7.4) using the shake-flask method. In two independent experiments vials were incubated at 25 °C and quantified by HPLC. Signals lower than the limit of quantification are indicated as <5 µM (lowest concentration used for calibration). Mean value and range are given. ^b^ Kinetic solubility using nephelometry. Mean value and range are given. * Singlicate measurement.

**Table 3:** Data collection and refinement statistics of the DYRK1A-ligand **11** complex.

PDB Accession Code	6T6A
	Data collection
beamline	Diamond Light Source, i04-1
wavelength (Å)	0.9200
Resolution ^a^ (Å)	46.23–2.80 (2.95–2.80)
space group	*C*2
cell dimensions	
a (Å)	244.9
b (Å)	65.4
c (Å)	148.1
α (deg)	90
β (deg)	115.2
γ (deg)	90
no. unique reflections ^a^	51,386 (7523)
Completeness ^a^ (%)	97.6 (98.5)
I/σI ^a^	7.5 (2.1)
R_merge_^a^	0.122 (0.535)
CC(1/2)	0.991 (0.757)
Redundancy ^a^	3.6 (3.7)
	Refinement
no. atoms in refinement (P/L/O) ^b^	11,336/48/436
R_fact_ (%)	20.5
R_free_ (%)	24.2
Bf (P/L/O) ^b^ (Å^2^)	49/55/39
rmsd bond ^c^ (Å)	0.010
rmsd deviation angle ^c^ (deg)	1.4
	Molprobity
Ramachandran favored	93.71
Ramachandran outlier	0.0

^a^ Values in brackets show the statistics for the highest resolution shells. ^b^ P/L/O indicate protein, ligand molecule, and other (water and solvent molecules), respectively. ^c^ rms indicates root-mean-square deviation.

## Supplementary Materials

The following are available online at https://www.mdpi.com/1420-3049/24/22/4090/s1, Figure S1: ^1^H NMR of 4-chlorocyclohepta[*b*]indol-10(*5H*)-one (**11**), Figure S2: ^13^C NMR of 4-chlorocyclohepta[*b*]indol-10(*5H*)-one (**11**), Figure S3: IR of 4-chlorocyclohepta[*b*]indol-10(*5H*)-one (**11**), Figure S4: MS (APCI+) of 4-chlorocyclohepta[*b*]indol-10(*5H*)-one (**11**), Figure S5: MS (APCI-) of 4-chlorocyclohepta[*b*]indol-10(*5H*)-one (**11**), Figure S6: HRMS/EIMS of 4-chlorocyclohepta[*b*]indol-10(*5H*)-one (**11**), Figure S7: HPLC gradient of 4-chlorocyclohepta[*b*]indol-10(*5H*)-one (**11**), Figure S8: HPLC isocratic of 4-chlorocyclohepta[*b*]indol-10(*5H*)-one (**11**).
