# Supplementary material for: [b]-Annulated Halogen-Substituted Indoles as Potential DYRK1A Inhibitors
Source: Molecules. 2019 Nov 13;24(22):4090. doi: 10.3390/molecules24224090 (PMC6891749; doi:10.3390/molecules24224090)

[*b*]-Annulated Halogen-Substituted Indoles as Potential DYRK1A Inhibitors

Supplementary Material

Christian Lechner, Maren Flaßhoff, Hannes Falke, Lutz Preu, Nadége Loaëc, Laurent Meijer, Stefan Knapp, Apirat Chaikuad, and Conrad Kunick

Spectra and chromatograms of 4-chlorocyclohepta[*b*]indol-10(5*H*)-one (11)

Table of contents:

Figure S1: ^{1}H-NMR of 4-chlorocyclohepta[*b*]indol-10(5*H*)-one (**11**) 2

Figure S2: ^{13}C-NMR of 4-chlorocyclohepta[*b*]indol-10(5*H*)-one (**11**) 3

Figure S3: IR of 4-chlorocyclohepta[*b*]indol-10(5*H*)-one (**11**) 4

Figure S4: MS (APCI+) of 4-chlorocyclohepta[*b*]indol-10(5*H*)-one (**11**) 5

Figure S5: MS (APCI-) of 4-chlorocyclohepta[*b*]indol-10(5*H*)-one (**11**) 6

Figure S6: HRMS/EIMS of 4-chlorocyclohepta[*b*]indol-10(5*H*)-one (**11**) 7

Figure S7: HPLC gradient of 4-chlorocyclohepta[*b*]indol-10(5*H*)-one (**11**) 8

Figure S8: HPLC isocratic of 4-chlorocyclohepta[*b*]indol-10(5*H*)-one (**11**) 9

Figure S1: ^{1}H-NMR spectrum of 11

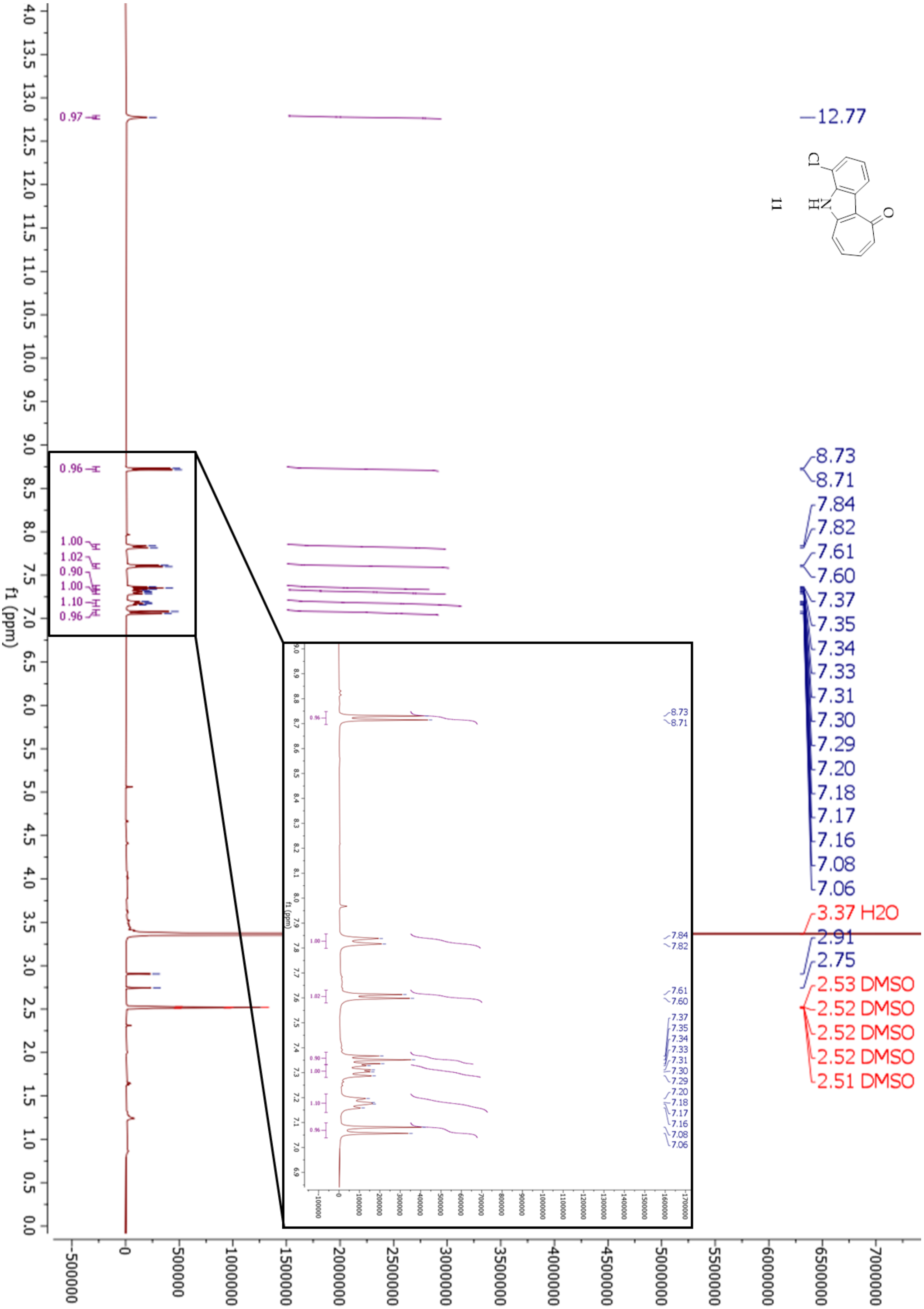

Figure S2: 13C-NMR spectrum of 11

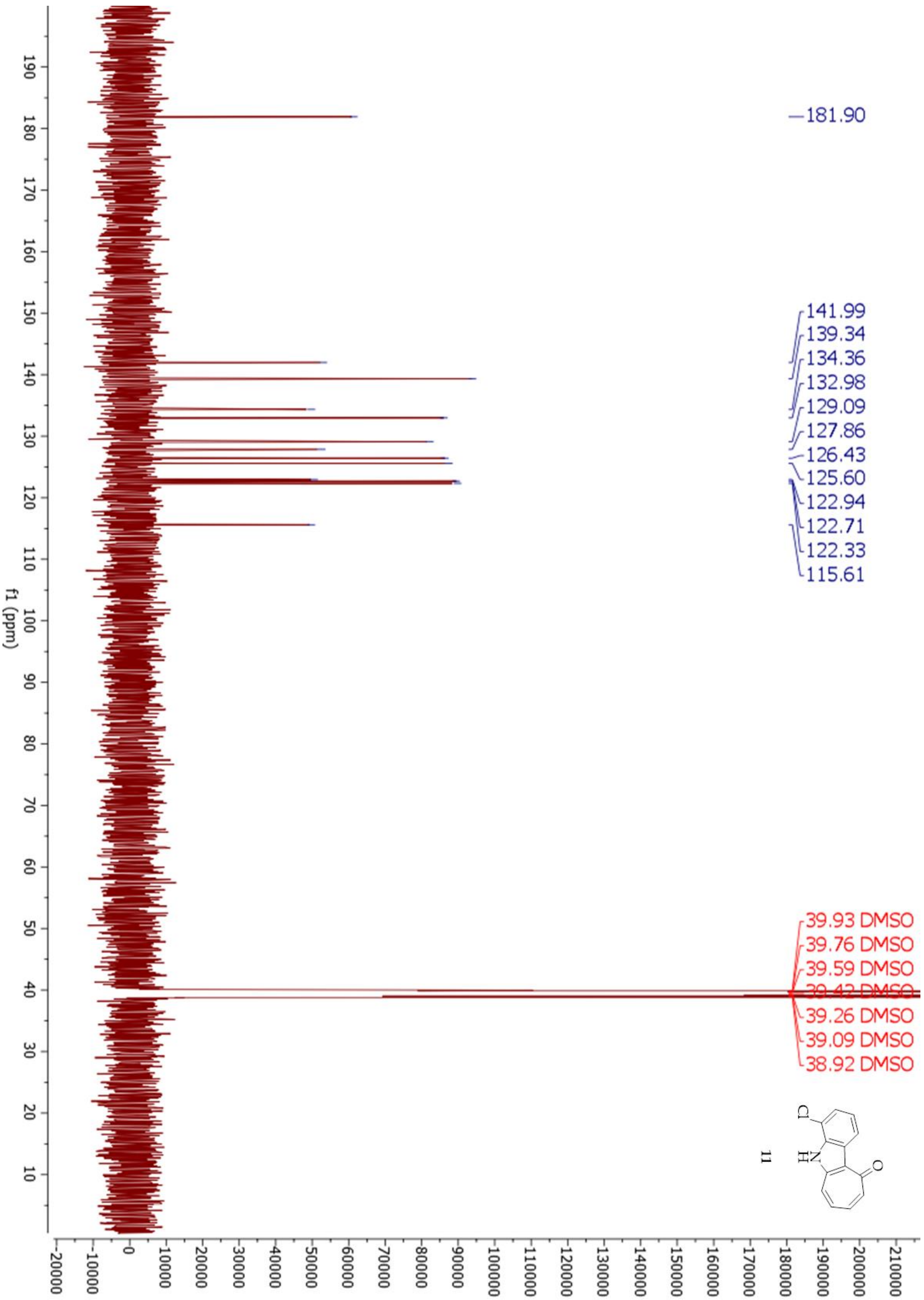

Figure S3: IR spectrum of 11

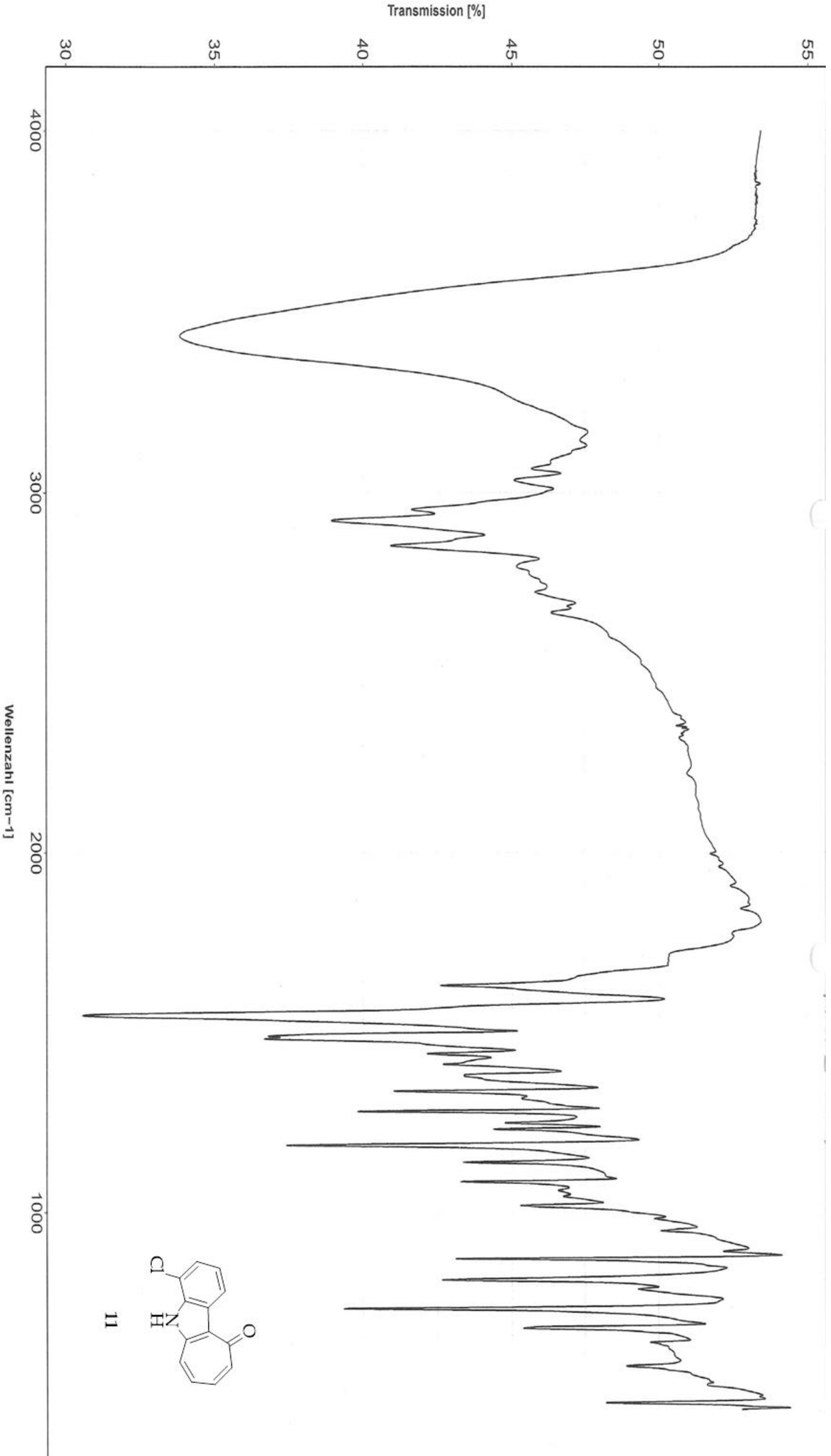

Figure S4: MS (APCI+) of 11

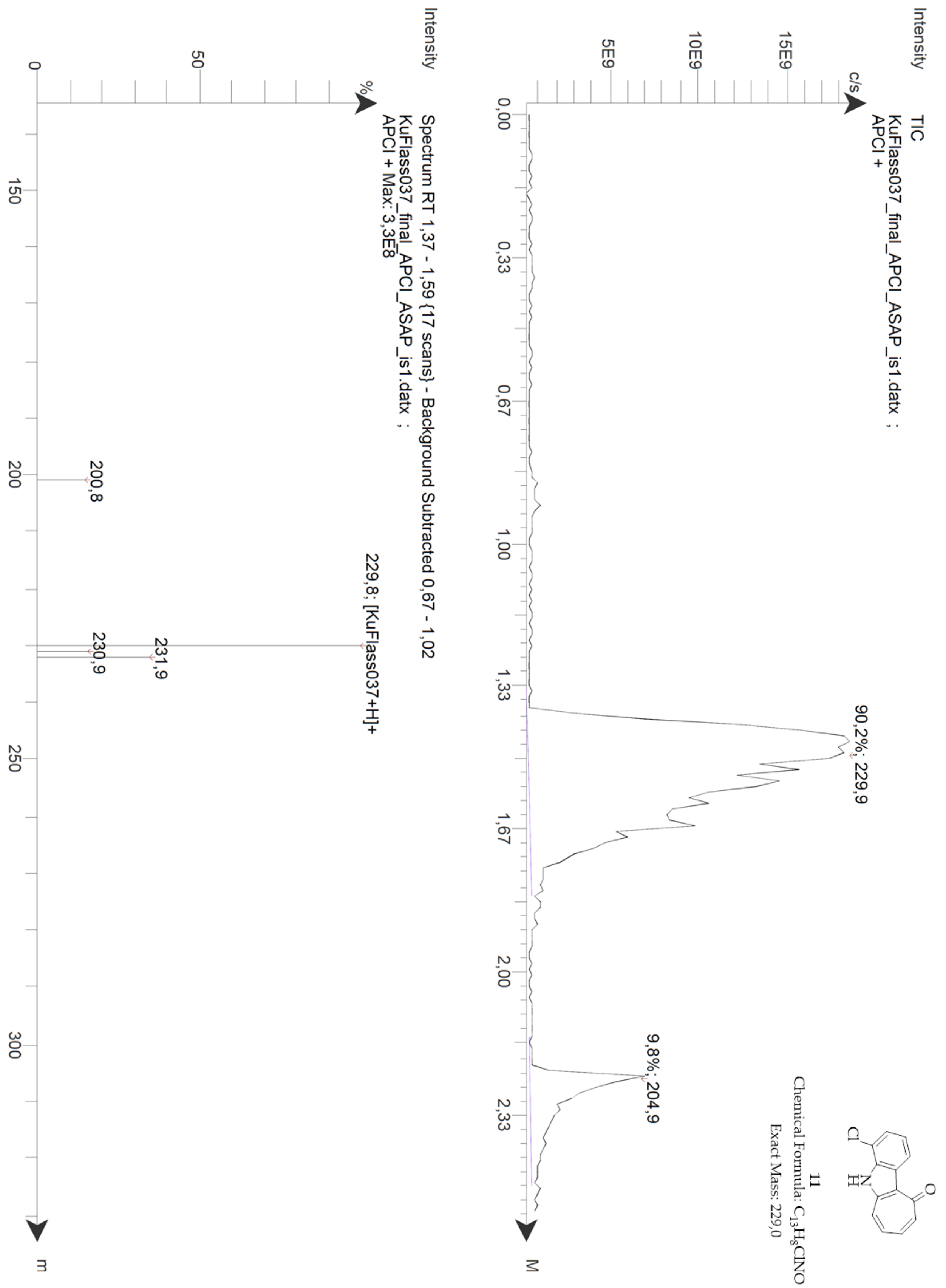

Figure S5: MS (APCI-) of 11

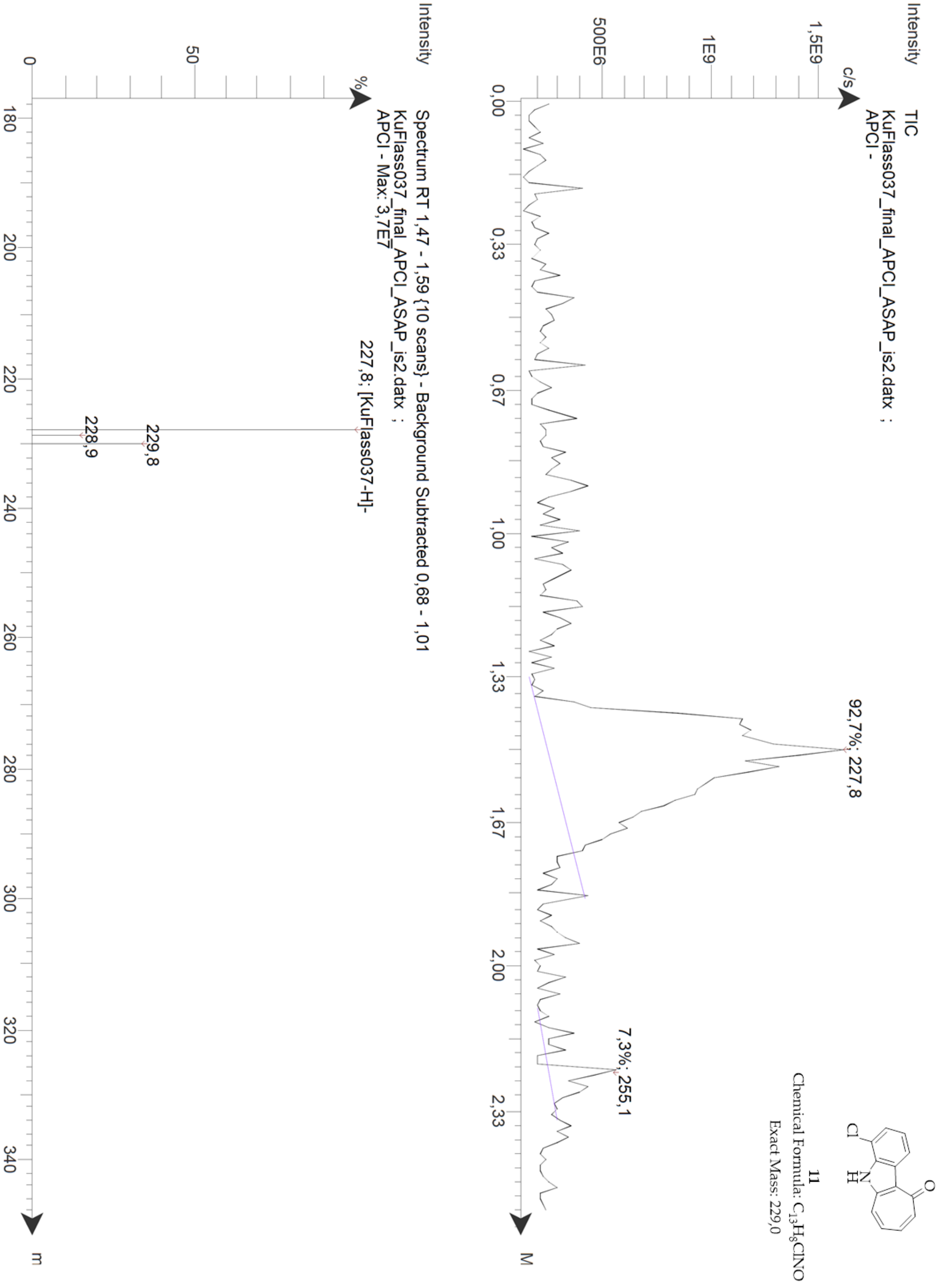

Figure S6: HRMS / full EIMS of 11

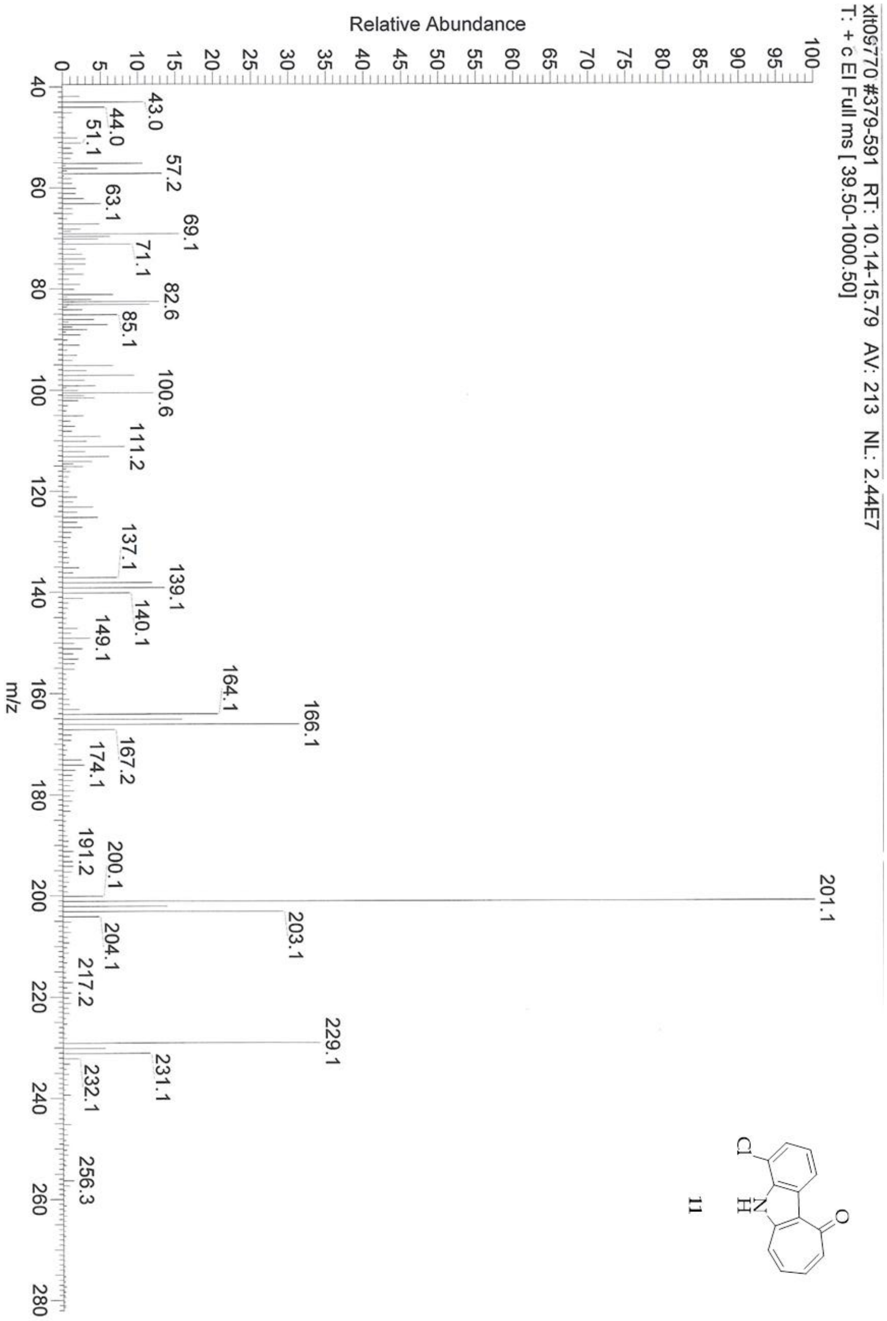

11

NOT ACQUIRING 28 Mar 2019 16:15 ELAPSED: END:

FILE:xlt09819 STUDY:pm AMOUNT: 0.00
OPER:cm CLIENT:KUNICK VOLUME: 0.00
SAMP:Flasshoff, pm zu XLT09770 INJ.VOL: 0.00
COMM:mw 229.02889 (geladen) / 229.02923 (exp.)

START

METHOD LIST: init(1)

RESTORE

FILE AUTOSAMP VIAL D:INLET ICL PROC. DS PROC. S/T/M DLY
1>xlt09819 userparam 1 userparam

ANALYSIS:

UCLR ULIST FILE: init

	UL2DATA	RESTORE		
1:	229.02920	0.0000	0.0000	0.00000
2:	229.02940	0.00000	0.00000	0.00000
3:	229.02942	0.00000	0.00000	0.00000
4:	229.02926	0.00000	0.00000	0.00000
5:	229.02928	0.00000	0.00000	0.00000
6:	229.02922	0.00000	0.00000	0.00000
7:	229.02907	0.00000	0.00000	0.00000
8:	229.02932	0.00000	0.00000	0.00000
9:	229.02922	0.00000	0.00000	0.00000
10:	229.02896	0.00000	0.00000	0.00000
11:	0.00000	0.00000	0.00000	0.00000
12:	0.00000	0.00000	0.00000	0.00000
13:	0.00000	0.00000	0.00000	0.00000
14:	0.00000	0.00000	0.00000	0.00000
15:	0.00000	0.00000	0.00000	0.00000
16:	0.00000	0.00000	0.00000	0.00000
17:	0.00000	0.00000	0.00000	0.00000
18:	0.00000	0.00000	0.00000	0.00000
19:	0.00000	0.00000	0.00000	0.00000
20:	0.00000	0.00000	0.00000	0.00000
LINK	NONE	NONE	NONE	NONE
SIZE	10.00000	0.00000	0.00000	0.00000
MEAN	229.02923	0.00000	0.00000	0.00000
SUM	2290.29234	0.00000	0.00000	0.00000
S.D.	0.00014	0.00000	0.00000	0.00000
MAX	229.02942	0.0000	0.00000	0.00000
MIN	229.02896	0.00000	0.00000	0.00000

ULIST:

Figure S7: Gradient HPLC of 11

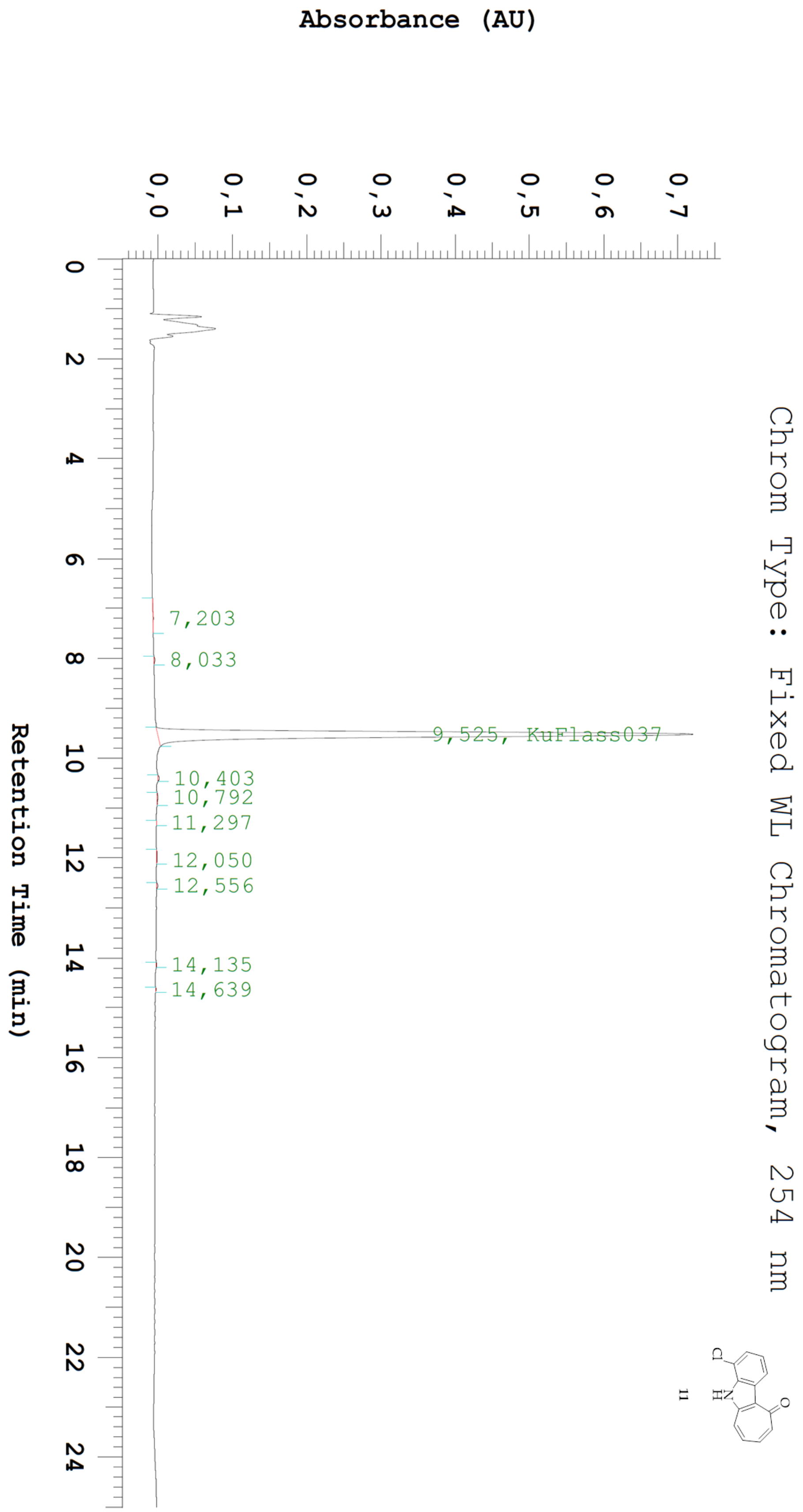

Figure S8: Isocratic HPLC of 11

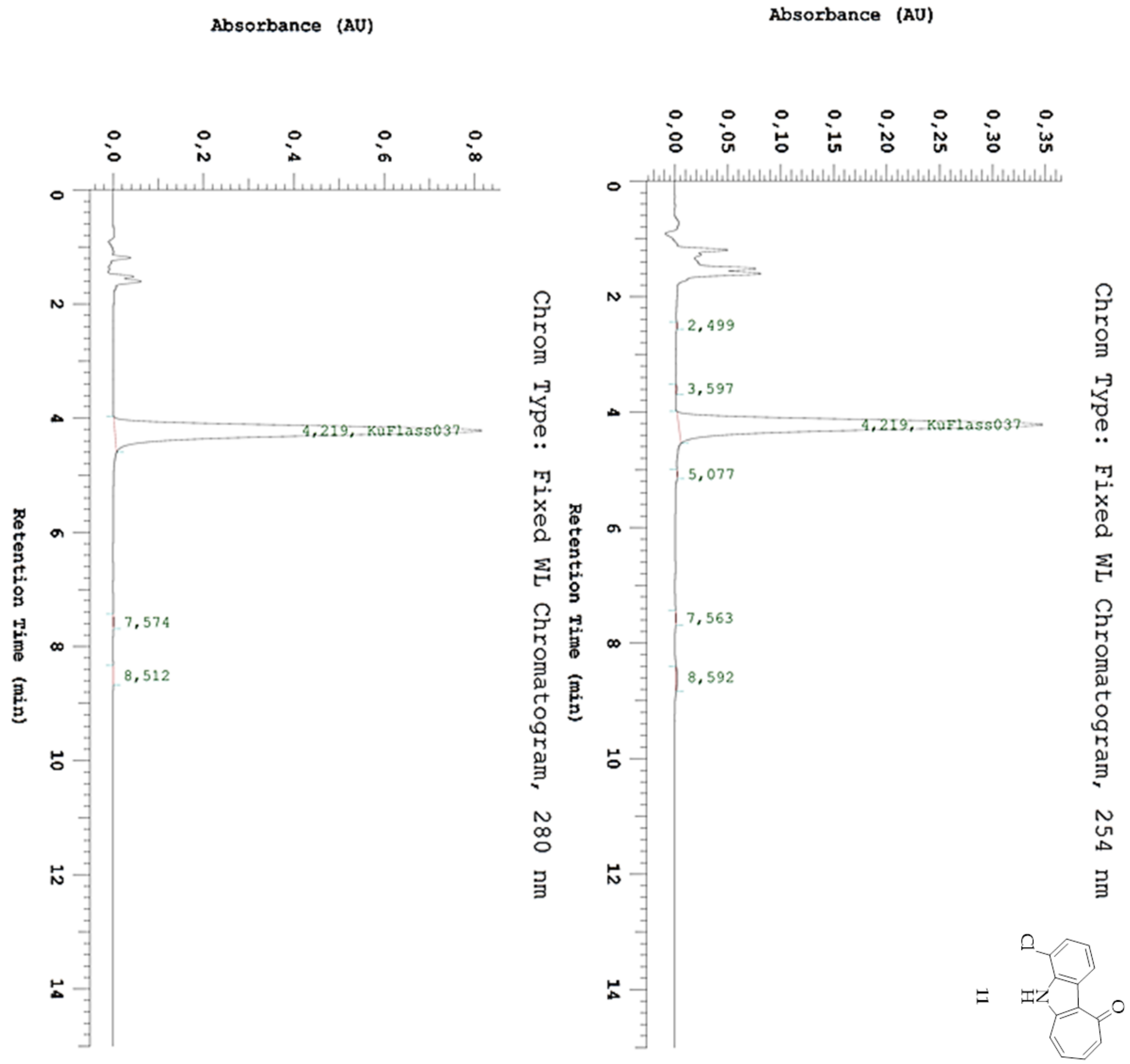

Supplement: Supplementary file 1 [file molecules-24-04090-s001.pdf]
